# Diabetes adversely affects phospholipid profiles in human carotid artery endarterectomy plaques[S]

**DOI:** 10.1194/jlr.M081026

**Published:** 2018-02-24

**Authors:** Mohamed A. Zayed, Fong-Fu Hsu, Bruce W. Patterson, Yan Yan, Uzma Naim, Malik Darwesh, Gayan De Silva, Chao Yang, Clay F. Semenkovich

**Affiliations:** Section of Vascular Surgery, Department of Surgery,* Center for Human Nutrition Washington University School of Medicine, St. Louis, MO; Department of Medicine,** and Division of Public Health Sciences, Washington University School of Medicine, St. Louis, MO; Department of Surgery,†† Washington University School of Medicine, St. Louis, MO; Department of Surgery,† Veterans Affairs St. Louis Health Care System, St. Louis, MO; Division of Endocrinology, Metabolism, and Lipid Research,§ Department of Medicine, Washington University, St. Louis, MO

**Keywords:** atherosclerosis, phospholipidomics, phosphatidylethanolamine, choline-ethanolamine phosphotransferase 1, arachidonic acid

## Abstract

Patients with diabetes are at higher risk of developing carotid artery stenosis and resultant stroke. Arachidonoyl phospholipids affect plaque inflammation and vulnerability, but whether diabetic patients have unique carotid artery phospholipidomic profiles is unknown. We performed a comprehensive paired analysis of phospholipids in extracranial carotid endarterectomy (CEA) plaques of matched diabetic and nondiabetic patients and analyzed mass spectrometry-derived profiles of three phospholipids, plasmenyl-phosphatidylethanolamine (pPE), phosphatidylserine (PS), and phosphatidylinositol (PI), in maximally (MAX) and minimally (MIN) diseased CEA segments. We also measured levels of arachidonic acid (AA), produced by pPE hydrolysis, and choline-ethanolamine phosphotransferase 1 (CEPT1), responsible for most pPE de novo biosynthesis. In paired analysis, MIN CEA segments had higher levels than MAX segments of pPE (*P* < 0.001), PS (*P* < 0.001), and PI (*P* < 0.03). MIN diabetic plaques contained higher levels than MAX diabetic plaques of arachidonoyl pPE38:4 and pPE38:5 and CEPT1 was upregulated in diabetic versus nondiabetic plaques. AA levels were relatively greater in MIN versus MAX segments of all CEA segments, and were higher in diabetic than nondiabetic plaques. Our findings suggest that arachidonoyl phospholipids are more likely to be abundant in the extracranial carotid artery plaque of diabetic rather than nondiabetic patients.

Phospholipid content in the arterial wall is altered by metabolic disorders such as diabetes and is an important contributor to the development and progression of atherosclerotic disease (1–3).

The “phospholipidomic code” representing the pattern of phospholipids in the setting of health and disease is becoming an area of interest in an effort to identify unique biochemical signatures that can influence atherosclerotic disease prevention and treatment in diabetic patients (3–5). In particular, ether lipids are known to have diverse biological effects on cellular and intracellular functions, and have an impact on tissue homeostasis and inflammation, as well as progression of cardiovascular disease (6, 7).

Ubiquitously expressed phosphatidylethanolamine (PE) accounts for approximately 20% of mammalian phospholipids (8). The majority of PEs are synthesized in the endoplasmic reticulum (ER) by the CDP-ethanolamine enzymatic pathway (Kennedy pathway) (9, 10). In the majority of mammalian tissue, the final step of PE synthesis is catalyzed by the essential enzyme, choline-ethanolamine phosphotransferase 1 (CEPT1) (11). Ether lipids derived from PE, such as plasmenyl-PE (pPE), are also synthesized by CEPT1 and are highly concentrated in vascular tissue and inflammatory cells (6, 7, 12). Although initial reports link pPE and CEPT1 to vascular tissue inflammation (3, 13, 14), how they are associated with human arterial plaque biology is largely unknown.

Diabetic patients are known to have more vulnerable carotid artery plaques that lead to a higher risk of resultant ischemic stroke (15). Prior reports show that the content of specific lipid subgroups is altered in the homogenates of heterogeneous whole carotid endarterectomy (CEA) plaques (2, 3, 14), as well as in plaques from other arterial beds (4, 16, 17). In this study, we build upon these prior observations by providing the first phospholipidomic analysis between maximally (MAX) and minimally (MIN) diseased CEA segments in diabetic and nondiabetic subjects with high-grade carotid artery stenosis.

## MATERIALS AND METHODS

### Human subjects and tissue processing

**Fig. 1.**
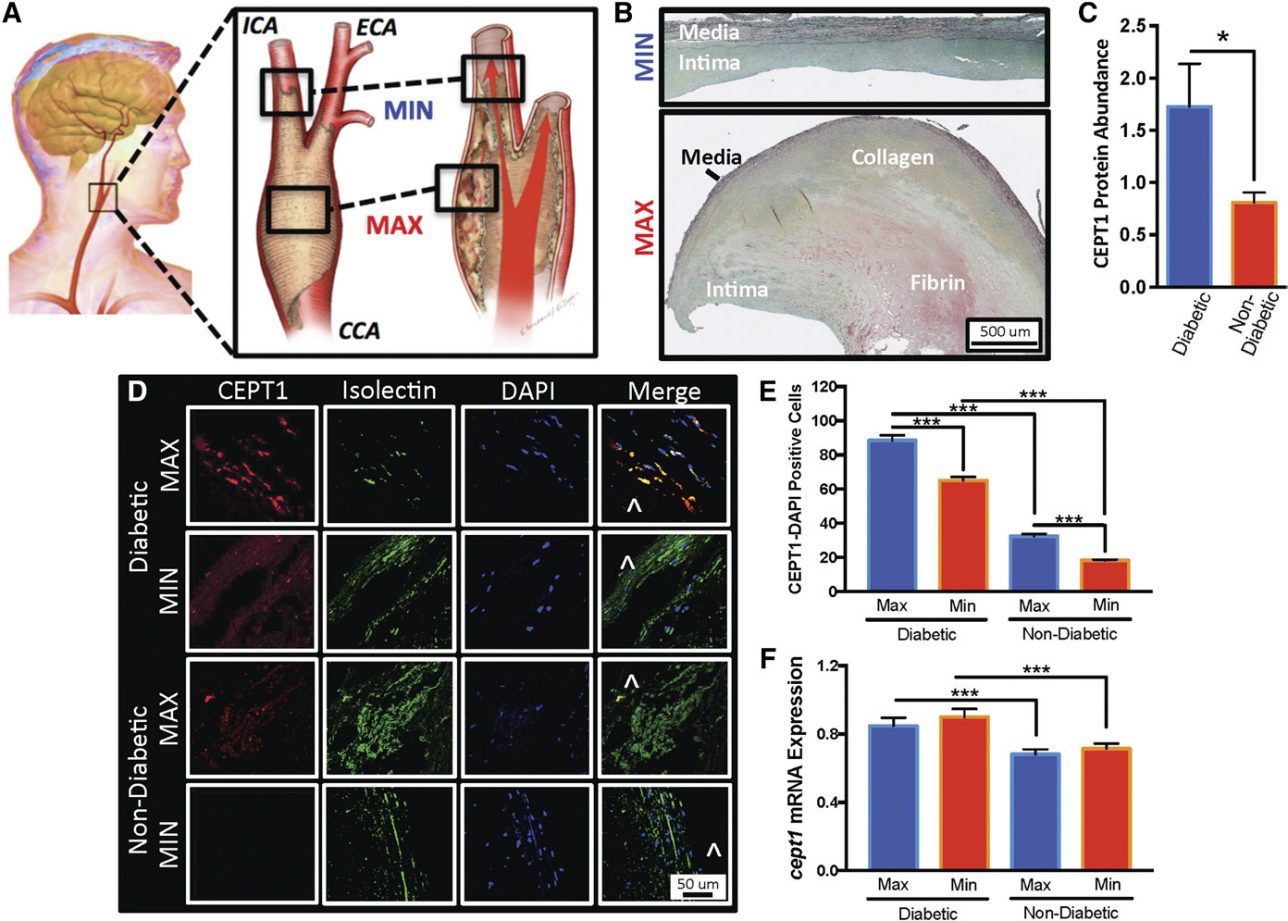
CEA plaques from diabetic subjects have higher CEPT1 expression. A: The extracranial common carotid artery (CCA) bifurcation is prone to atherosclerosis. Within the plaque there are segments of MAX disease at the level of the carotid bifurcation and segments of MIN disease at the distal edge of the plaque within the lumen of the internal carotid artery (ICA). ECA, external carotid artery. B: Pentachrome staining of CEA plaque demonstrates that MIN diseased segments retain an intima layer surrounded by a relatively intact internal elastic lamina and partial media layer. The MAX segment has a grossly abnormal thick intima layer with areas of necrosis, intra-plaque hemorrhage, non-intact internal elastic lamina layer, and a thinner medial segment. Black, elastic fibers; yellow, collagen fibers; blue, mucin; bright red, fibrin. C: Quantified Western blot analysis demonstrates higher CEPT1 protein expression in CEA plaques of diabetic subjects (n = 11) compared with nondiabetic (n = 7) subjects. D: Immunofluorescent staining demonstrates more CEPT1-DAPI-positive cells in the superficial intima of MAX segments. Isolectin staining for ECs and smooth muscles, DAPI staining for nuclei, and ^ indicates vessel lumen. E: Quantitative analysis of CEPT1-positive cells in five random of 20× images demonstrates higher CEPT1 content in MAX and MIN diseased segments of diabetic subjects, and also higher content in MAX segments of diabetic (n = 8) and nondiabetic (n = 17) subjects. F: Relative expression of *cept1* was higher in diabetic MAX and MIN diseased segments. Error bars = SEM, *P < 0.05 and ***P < 0.001.

Forty-nine subjects with >70% stenosis in the extracranial carotid artery bifurcation participated in this study, which was approved by the local Human Research Protection Office. Using an IRB-approved protocol, all subjects provided research consent to allow collection of intraoperative CEA plaque specimens at the time of their CEA. In the operating room, CEA plaques were removed en bloc from the subject’s carotid bifurcation, and immediately sectioned into MAX (the segment that is at the level of the carotid bifurcation) and MIN diseased segments (the segment that is at the plaque periphery distal edge in the internal carotid artery; Fig. 1A). The CEA plaque segments were independently inspected to confirm that MAX diseased segments had American Heart Association type IV–VIII atherosclerotic plaques, and MIN diseased segments had American Heart Association type I–III atherosclerotic plaques (Fig. 1) (18).

### CEA protein and lipid extraction

Plaque segments were immersed in cold hypotonic nondetergent-based lysis buffer [1 M NaHCO_3_, 1 M sucrose, 1.5 M NaN_3_, 0.1 M PMSF, and Protease Inhibitor Cocktail Set III (Calbiochem, San Diego, CA)] for 30 min. All specimens were then homogenized with a high-speed rotational power tissue homogenizer (Glas-Col, Terre Haute, IN). Homogenized samples underwent centrifugation (4,697 *g* for 5 minutes), and the supernatants were collected and standardized relative to protein concentration using a colorimetric Bradford protein concentration assay (Bio-Rad, Hercules, CA). Supernatant aliquots were obtained for each sample and a set quantity of homologous nonnaturally occurring phospholipid internal standard species was added to each aliquot. This internal standard cocktail included 1,2-dimyristoyl-*sn*-glycero-3-phosphocholine [phosphatidylcholine (PC) 14:0/14:0], dimyristoyl-*sn*-glycero-3-phosphoethanolamine (PE 14:0/14:0), dimyristoyl-*sn*-glycero-3-phosphochoserine [phosphatidylserine (PS) 14:0/14:0], dimyristoyl-*sn*-glycero-3-phosphoglycerol [phosphatidylglycerol (PG) 14:0/14:0], dipalmitoyl-*sn*-glycero-3-phosphoinositol [phosphatidylinositol (PI) 16:0/16:0], and *N*-lauroyl-D-*erythro*-sphingosine [ceramide (Cer) d18:1/12:0]. Samples were mixed with lipid extraction buffer [2:2 (v/v) chloroform/methanol] and the organic phase was collected. Extracts were then dried under nitrogen and reconstituted in methanol with 0.25% NH_4_OH (29%) (19).

### Electrospray ionization mass spectrometry

Lipid extracts from MAX and MIN diseased samples obtained from 17 patients (10 diabetic and 7 nondiabetic; supplemental Table S1) were analyzed by direct injection electrospray ionization mass spectrometry using a Thermo Vantage triple-quadruple mass spectrometer (San Jose, CA) and an Accela 1250 UPLC system operated via an Xcalibur operating system. From each extract, 10 ul were loop injected into the electrospray ion source. The mass spectrometer skimmer was set at ground potential; the electrospray needle was set at 3.0 kV for positive-ion and 2.5 kV for negative-ion mode operation; and the temperature of the heated capillary was set at 300°C. Argon was used as the collision gas for linked scan collision-induced dissociation tandem mass spectrometry. Precursor-ion scan (PIS) and neutral loss scan (NLS) were used to profile PC (PIS of 184) as [M+H]^+^ ions, and PE (PIS of 196), PI (PIS of 241), PG (PIS of 153), PS (NLS of 87), and Cer (NLS of 256) as [M-H]^−^ ions, under each optimal collision energy and collision gas pressure. Supplemental Figs. S1 and S2 provide an example of two sets of linked scan spectra of the phospholipid families analyzed in MAX and MIN diseased CEA segments from a single nondiabetic human subject.

Structures for all phospholipids were identified using a multiple-stage linear ion-trap that was operated at a low energy collision-induced dissociation and high-resolution mass spectrometry. Phospholipid structural assignments were made as previously described (20–29), and as summarized in supplemental Tables S2–S10. Supplemental Figs. S3–S8 provide product-ion spectra that were used to produce structural determinations for each phospholipid group.

### Mass spectrometry phospholipid data analysis

The mass spectrometry-derived lipid mass spectrum for each sample was averaged over time, and the average background spectrum was subtracted from it. The net sample spectrum were described as a set of signal intensities of the predefined phospholipid species whose isotopologue distribution patterns for m+0 through m+4 natural abundance isotopologues were determined using a custom algorithm in MATLAB (Mathworks, Inc., Natick, MA). Absolute lipid concentration quantitation was obtained by deriving the ratio of the signal intensity of each species against the known quantity of the homologous nonnaturally occurring internal standard species added to the lipid extract samples. This analysis included 50 PC-related species [including 23 PCs (supplemental Table S2), 16 alkyl ether PCs (aPCs; supplemental Table S3), and 11 SMs (supplemental Table S4)], 25 PS species (supplemental Table S5), 22 PE-related species [including 11 PEs (supplemental Table S6) and 11 pPEs (supplemental Table S7)], 13 PG species (supplemental Table S8), 16 PI species (supplemental Table S9), and 7 Cer species (supplemental Table S10).

### Western blotting and ELISA

Lysed tissue homogenates from MAX and MIN diseased samples (from the matched 10 diabetic and 7 nondiabetic human subjects that underwent mass spectrometry lipidomic analysis; supplemental Table 1) were separated using a gradient-gel SDS-PAGE, and transferred to a PVDF membrane for Western blotting using anti-human CEPT1 antibody (Abcam). Lysed tissue homogenates from an additional 11 subjects (5 diabetic and 6 nondiabetic, age and comorbidity matched; supplemental Table S11) were similarly blotted for human anti-cytosolic phospholipase A_2_ (cPLA_2_) antibody (Abcam, ab198898), anti-calcium-independent PLA_2_ (iPLA_2_) antibody (Abcam, ab103258), and anti-GAPDH antibody (Cell Signaling, #2118). Bands were quantified by densitometry. Using the same 11 subject samples evaluated for cPLA_2_ protein expression, the levels of nonesterified arachidonic acid (AA) in MAX and MIN diseased segments were evaluated using a competitive ELISA assay (Aviva Systems Biology, OKEH02583) according to manufacturer’s instructions.

### Quantitative real-time PCR

Freshly collected MAX and MIN diseased CEA segments from 23 subjects (8 diabetic and 15 nondiabetic, age and comorbidity matched; supplemental Table S12) were submerged in liquid nitrogen and crushed into a fine powder using a mortar and pestle. Crushed samples were then placed in 1 ml TRIZOL (Thermo Fisher), and RNA was extracted using an RNeasy Mini kit (Qiagen). Following extraction, RNA concentration and integrity were evaluated using an Agilent 2100 Bioanalyzer RNA Nano kit (Agilent) with RIN <8. One microgram of RNA per sample was converted to cDNA using an iScript cDNA Synthesis kit (Bio-Rad). cDNA concentration was evaluated using NanoDrop (Thermo Fisher ND-ONE-W) and adjusted to a standard concentration across all samples. Quantitative PCR was carried out using the SYBR Green PCR Master Mix (Applied Biosystems) one-step RT-PCR protocol using *cept1* forward (5′-AGG TGG TCC TCCAAT CAC TG-3′) and reverse (5′-TGG CAA ACG TAT GTT TCT GG-3′) primers. Amplification was performed using StepOnePlus real-time PCR system (Thermo Fisher) to determine relative changes in amplification cycle between different MAX and MIX diseased CEA specimens.

### Confocal microscopy and immunohistochemistry

MAX and MIN diseased segments were serially dehydrated in 15% and 30% sucrose solutions. CEA segments were then embedded in OCT and sectioned at 10 μm sections. Following fixation with 4% paraformaldehyde, tissue sections were denatured, permeablized with 1% Triton/PBS solution, and blocked with 1% BSA, 0.2% milk powder, 0.3% Triton X-100 solution. CEA sections were then stained with Pentachrome, as previously described (30), or with anti-CEPT1 antibody (1:50; Proteintech, Rosemont, IL), followed by labeling with Alexa Fluor 594-conjugated secondary antibody (1:250; Invitrogen, Waltham, MA). Tissue sections were counterstained with DAPI and with Alexa Fluor 488-conjugated *Griffonia simplicifolia* isolectin-1-B4 (1:100; Invitrogen). Representative images of CEPT1-stained CEA tissue sections were obtained at 60× magnification using a Nikon A1Rsi confocal microscope. Using a DFC 3000 G Leica Microsystems inverted fluorescent microscope, 30 random 20× images were obtained of the superficial intima of MAX and MIN diseased specimens of three diabetic and three nondiabetic patients. Quantitative assessments of CEPT1- and isolectin-1-B4-positive cells in the superficial intima were performed using Image J software.

### Statistics

A nonparametric Mann-Whitney U test was used to evaluate differences in age, demographics, and medications between diabetic and nondiabetic subjects who provided CEA plaque specimens. Mass spectrometry descriptive analysis was performed to evaluate heat map relative fold differences in phospholipid mean absolute quantities. A nonparametric Wilcoxon two-sample test was used to evaluate differences between phospholipid groups between MAX and MIN diseased segments, as well as diabetic and nondiabetic patient groups. Further subgroup analysis was performed using a univariate signed-rank test to evaluate differences in phospholipid classes between MAX and MIN diseased segments in all patients combined, or selectively among diabetic and nondiabetic patients. Similarly, a univariate signed-rank test was used to evaluate differences in specific phospholipid species in all patients combined, or selectively among diabetic and nondiabetic patients. A two-tailed Student’s *t*-test was used to evaluate relative differences in specific protein and mRNA expression in MAX and MIN diseased CEA segments from diabetic and nondiabetic patients. For all statistical tests, *P* < 0.05 was considered statistically significant and was not adjusted for multiple comparisons.

## RESULTS

### Patient clinical demographics

Biochemical and molecular analysis of MAX and MIN diseased human CEA plaque segments was performed using specimens collected from 49 human subjects (21 diabetic and 28 nondiabetic). Comparative analysis of subject demographics and medication profiles revealed no difference in age groups, cardiovascular risk factors, or prevalence of symptomatic carotid artery stenosis prior to CEA (**Table 1**). As expected, diabetic subjects were more likely to be clinically obese (BMI >30; *P* < 0.001) and taking medications such as β-blockers, anti-platelets, and insulin (*P* < 0.05; Table 1). Matched human subjects were used for each subgroup analysis (supplemental Tables S2–S4).

**TABLE 1.** Demographics of patients in cohort 1

	Nondiabetic	Diabetic	*P*
Age			
50–60 (%)	18	10	0.42
61–70 (%)	29	48	0.18
71–80 (%)	43	33	0.51
81–90 (%)	11	10	0.94
Demographics			
Gender (n)	M28/F8	M21/F8	0.49
BMI ≥30 (%)	14	67	<0.001*^a^*
Current Smoker (%)	11	29	0.11
Hypertension (%)	79	95	0.10
Hyperlipidemia (%)	82	90	0.42
CAD (%)	43	48	0.75
Stroke (%)	32	29	0.79
Medications			
Antiplatelet (%)	100	76	0.02*^a^*
Beta-blocker (%)	25	76	<0.001*^a^*
Statin (%)	82	81	0.92
Insulin (%)	0	33	<0.001*^a^*

a Significance with a nonparametric Mann-Whitney U test. CAD, coronary artery disease.

### CEPT1 expression is elevated in diabetic CEA specimens

Our group recently demonstrated that skeletal muscle CEPT1 affects insulin sensitivity and lipid metabolism in diet-induced diabetes (31). To determine whether CEPT1 is similarly altered in the carotid arteries of diabetic human subjects, we evaluated CEA plaques (**Fig. 1A, B**) of 17 subjects and observed a 53% increase in CEPT1 protein expression in diabetic specimens (*P* < 0.05; Fig. 1C). Immunofluorescent microscopy detected scattered CEPT1 tissue staining in the intima of MAX and MIN diseased segments (Fig. 1D). There was more CEPT1 staining in the superficial intima of MAX and MIN segments of diabetic subjects (Fig. 1E; *P* < 0.001), and even more staining in MAX segments compared with diseased MIN segments. Expression patterns were further confirmed in an additional 23 human subjects (7 diabetic and 16 nondiabetic), with higher *cept1* mRNA expression in both the MAX and MIN diseased CEA segments of diabetic subjects (*P* < 0.001; Fig. 1F).

### Content of pPEs and PSs is increased in MIN diseased CEA segments

The terminal enzyme of the Kennedy pathway is CEPT1, and it is responsible for the de novo biosynthesis of the majority of phospholipids in mammalian tissue, including pPEs (9, 10). Because we observed higher *cept1* expression in CEA plaque of diabetic subjects, we next evaluated the potential consequences of this on tissue phospholipid content. Paired mass spectrometry analysis of all major phospholipid groups revealed a significant relative increase in the absolute quantities of pPEs and PSs in MIN segments of diabetic subjects (**Table 2**; *P* = 0.001 and *P* < 0.001, respectively). The relative absolute quantity of PIs was only increased in MIN segments of nondiabetic subjects (*P* = 0.03), and pPEs were only increased in the MIN segments of diabetic subjects (Table 2; *P* = 0.01). No significant differences were observed in the relative absolute quantities of PC, aPC, SM, PE, Cer, or PG groups between MAX and MIN segments of diabetic and nondiabetic subjects (Table 2).

**TABLE 2.** Paired phospholipid analysis between MAX and MIN diseased CEA segments in matched nondiabetic and diabetic subjects

Lipid Family	All			Nondiabetic			Diabetic		
	MAX (nmol/ug)	MIN (nmol/ug)	*P*	MAX (nmol/ug)	MIN (nmol/ug)	*P*	MAX (nmol/ug)	MIN (nmol/ug)	*P*
PC	0.63	0.75	0.24	0.67	0.97	0.11	0.61	0.59	1
aPC	0.3	0.31	0.85	0.33	0.44	0.3	0.28	0.21	0.28
SM	1.51	1.57	0.68	1.57	2.02	0.38	1.46	1.26	1
PE	0.23	0.24	0.64	0.25	0.23	0.81	0.21	0.24	0.43
pPE	0.3	0.47	0.001*^a^*	0.3	0.42	0.11	0.29	0.51	0.01*^a^*
PI	0.14	0.19	0.05*^a^*	0.14	0.22	0.03*^a^*	0.14	0.17	0.56
Cer	0.06	0.05	0.49	0.06	0.07	0.94	0.05	0.03	0.32
PS	0.19	0.44	<0.001*^a^*	0.17	0.38	0.03*^a^*	0.2	0.5	<0.01*^a^*
PG	0.02	0.02	0.42	0.02	0.02	0.3	0.02	0.02	0.97

a Significance with a univariate signed-rank test.

### Arachidonoyl pPEs are more abundant in MIN diseased CEA segments

**Fig. 2.**
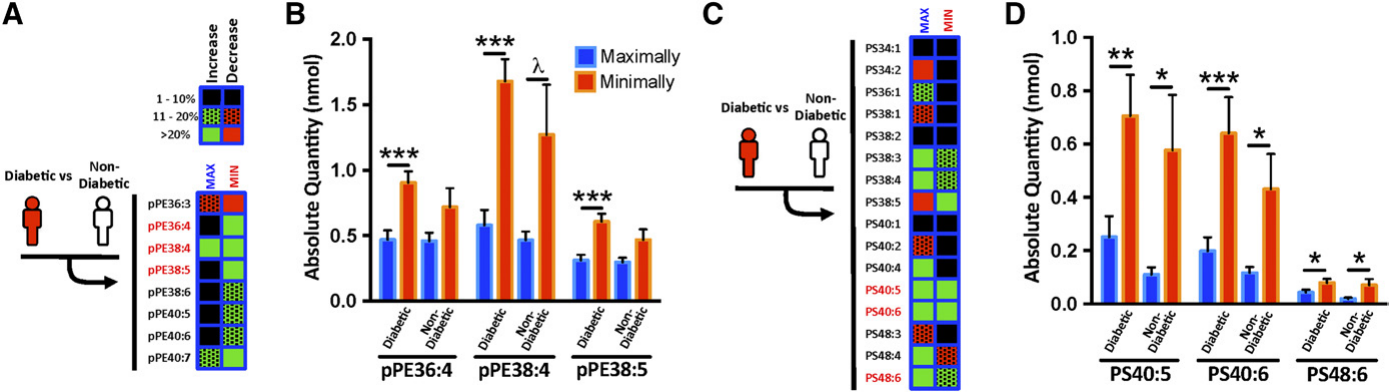
Differential pPE and PS content in MAX and MIN diseased CEA segments of diabetic and nondiabetic subjects. A: Heat map analysis of mass spectrometry lipidomics of MAX and MIN diseased CEA segments from diabetic (n = 10) versus nondiabetic (n = 7) subjects demonstrates increased (green) and decreased (red) content of specific pPE species. Among pPEs, the highest (>20%) relative differences were observed in pPE36:4, pPE38:4, and pPE38:5. B: The absolute quantities of these pPE species are higher in MIN segments compared with MAX segments. C: Heat map analysis of MAX and MIN segments from diabetic versus nondiabetic subjects also demonstrate increased and decreased content of specific PS species. Among PSs, the highest relative differences were observed in PS40:5, PS40:6, and PS48:6. D: The absolute quantity of these PS species is also higher in MIN segments. Error bars = SEM, λ *P* = 0.06, *P < 0.05, **P < 0.01, and ***P < 0.001.

To determine which specific pPE and PS species were most notably altered, we performed a phospholipid subgroup heat map analysis in MAX and MIN diseased segments of diabetic versus nondiabetic subjects (**Fig. 2A, C**). This revealed a >20% relative change in the absolute quantities of specific pPEs (pPE36:4, pPE38:4, pPE38:5, and pPE40:7; Fig. 2A) and PSs (PS38:3, PS38:5, PS40:4, PS40:5, PS40:6, PS48:4, and PS48:6; Fig. 2C). Compared with MAX, the MIN segments of diabetic subjects demonstrated higher amounts of arachidonoyl species, 1-(1Z-octadecenyl)-2-arachidonoyl-*sn*-glycero-3-phosphoethanolamine (pPE38:4; 32% increase; *P* < 0.001) and 1-(1Z,9Z-octadecedienyl)-2-arachidonoyl-*sn*-glycero-3-phosphoethanolamine (pPE38:5; 30% increase; *P* < 0.001; Fig. 2B). Similarly, the absolute quantities of PS40:5, PS40:6, and PS48:6 were also increased in MIN segments of diabetic subjects (Figs. 2D). The same arachidonoyl pPE and PS species were also abundant in MIN diseased segments of CEA plaques from asymptomatic subjects (who had no stroke symptoms prior to CEA; supplemental Fig. S9).

### AA is more abundant in MIN diseased CEA plaque segments

**Fig. 3.**
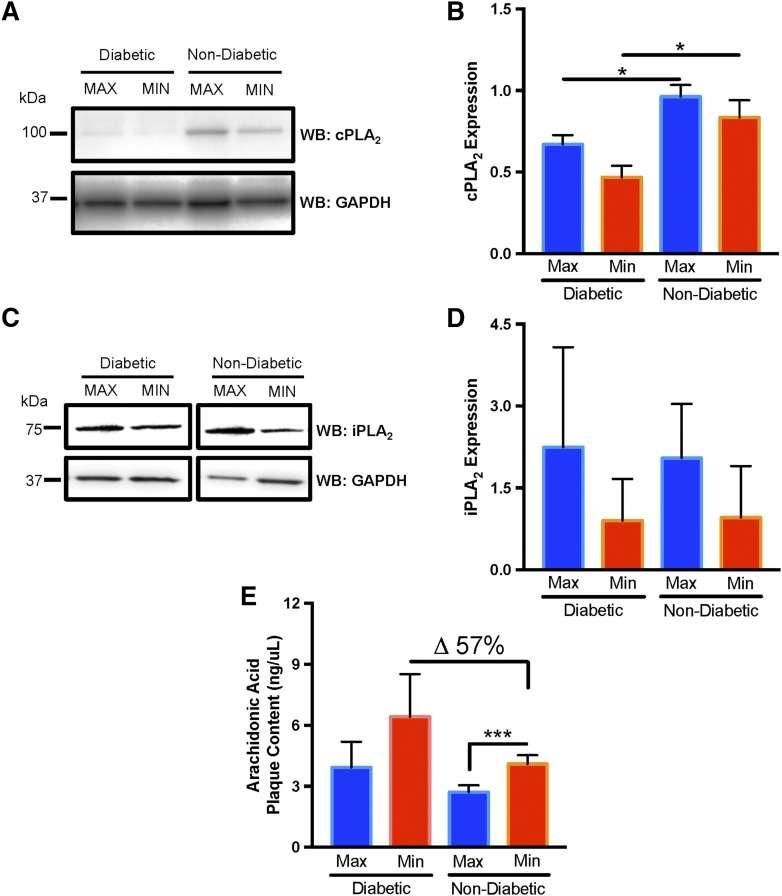
Differential content of cPLA_2_ and AA in CEA plaque segments of diabetic subjects. A: Representative Western blot (WB) demonstrates higher cPLA_2_ abundance in the MAX and MIN diseased CEA segments of nondiabetic subjects. GAPDH is a loading control between lanes. B: Quantitative Western blot cPLA_2_ band intensity, normalized to GAPDH, demonstrated higher cPLA_2_ in MIN and MAX diseased segments of nondiabetic subjects (n = 5) compared with diabetic subjects (n = 6). C: Representative Western blot demonstrates no difference in iPLA_2_ abundance in the MAX and MIN diseased CEA segments of diabetic and nondiabetic subjects. D: Quantitative Western blot iPLA_2_ band intensity, normalized to GAPDH, demonstrates no difference in iPLA_2_ in MIN and MAX diseased segments of diabetic (n = 6) and nondiabetic subjects (n = 5). E: ELISA analysis of AA content in MAX and MIN diseased CEA segments demonstrates higher AA content in MIN segments in both diabetic (n = 7) and nondiabetic (n = 5) patients. Error bars = SEM, *P < 0.05 and ***P < 0.001.

Atherosclerosis increases group IVA cPLA_2_ expression and activation in the artery media (32), which results in arachidonoyl phospholipid hydrolysis and the release of AA (33). Increased AA potentiates tissue inflammation (34) and is argued to contribute to atheroprogression in the setting of diabetes (35, 36). To evaluate the potential contributions of this in CEA plaque of diabetic subjects, we evaluated cPLA_2_ protein abundance in MAX and MIN segments. We observed slightly higher cPLA_2_ in MAX segments of both diabetic and nondiabetic subjects (**Fig. 3A, B**); however, both MAX and MIN segments from nondiabetic patients contained more cPLA_2_ (*P* < 0.05; Fig. 3B). We observed no difference in group VIA/B iPLA_2_ in both MAX and MIN segments, suggesting that phospholipid reacylation via this enzyme was not as affected (Fig. 3C, D).

Because the content of both arachidonoyl pPEs and cPLA_2_ levels in CEA plaque appeared to be altered relative to disease severity and the presence of diabetes, we next explored whether AA content was also different between MIN and MAX diseased CEA segments. Similar to arachidonoyl pPEs, AA content was relatively higher in MIN segments (Fig. 3E), and AA content was notably higher in the MIN segments of diabetic subjects (57% higher than MIN segments of nondiabetic subjects).

## DISCUSSION

How diabetes can alter the phospholipidome of carotid artery plaque to affect stroke outcomes is poorly understood and has direct translational relevance. Lipid-lowering agents are thought to decrease carotid atheroprogression and decrease the risk of stroke (37, 38). However, despite more prevalent use of statins and tighter glycemic control, diabetic patients are still six times more likely to suffer from a disabling stroke (15), an observation that is difficult to reconcile with current treatment guidelines for diabetic patients with progressive asymptomatic carotid artery stenosis (39, 40). To provide novel insights into the complex relationship between diabetes and tissue phospholipogenesis, we tested the hypothesis that diabetic subjects have altered CEPT1 plaque expression and phospholipid content in variably diseased (MIN vs. MAX) carotid artery plaque segments.

Our results confirm that MIN and MAX diseased CEA segments have different phospholipidomic profiles. To our knowledge, our group is the first to evaluate the phospholipidomic content and metabolism of variably diseased CEA segments from matched human subjects. This is highly informative, because the angle, curvature, and width across the carotid bifurcation contribute to differential intimal shear stress leading to heterogeneous plaque deposition in the carotid intima with areas of variable hemorrhage, inflammation, and necrosis (41). Prior studies confirm that diabetic subjects are more likely to have vulnerable CEA plaque features and altered lipid content (2, 42). However, the majority of these human studies do not account for the known heterogeneous nature of CEA plaques, and are limited by the lack of relatively “less-diseased” specimen controls.

The role of CEPT1 in human plaque biology is currently completely unknown. As the key terminal enzyme of the Kennedy pathway (9, 43), it is responsible for the biosynthesis of PCs, PEs, and pPEs. Our findings are the first to demonstrate that both CEPT1 protein and *cept1* mRNA expression are significantly increased in MIN diseased CEA segments of diabetic subjects. We also observed that MIN diseased CEA segments contained the highest content of arachidonoyl pPEs (pPE38:4 and pPE38:5). On the other hand, MAX diseased CEA segments demonstrated both lower CEPT1 expression and lower arachidonoyl pPE plaque content. The observed correlations between CEPT1 expression and pPE content in MIN and MAX diseased CEA segments suggests that CEPT1 is an important mediator of plaque phospholipogenesis. Our observations are also consistent with recent reports that relate a complex interplay between intima lipid infiltration and inflammation (44, 45), which leads to deleterious/destabilizing effects at the plaque edge that promote plaque rupture and thrombosis (46, 47).

These findings also add to the growing body of evidence that diabetes affects CEPT1 expression and its role in phospholipid metabolism (31, 48). In skeletal muscles, diet-induced diabetes increases murine CEPT1 expression, and weight loss in obese human subjects decreases CEPT1 expression (31). Mice with a skeletal muscle CEPT1 deficiency have increased insulin sensitivity and altered muscle tissue lipid compartmentalization with diet-induced obesity. Similarly, CEPT1 knockdown in the liver (where it is naturally highly expressed) downregulates PPARα-dependent gene expression, while CEPT1 overexpression in the liver leads to increased PPARα-induced gene expression (48). Thus, in the setting of diabetes, it is evident that CEPT1 generates important phospholipid ligands that can increase tissue phospholipid content and oxidative stress (49). Although it is yet to be determined to what extent CEPT1-mediated phospholipogenesis occurs in human plaque, our data suggests that it is perhaps more active in the arterial plaque of diabetic subjects.

**Fig. 4.**
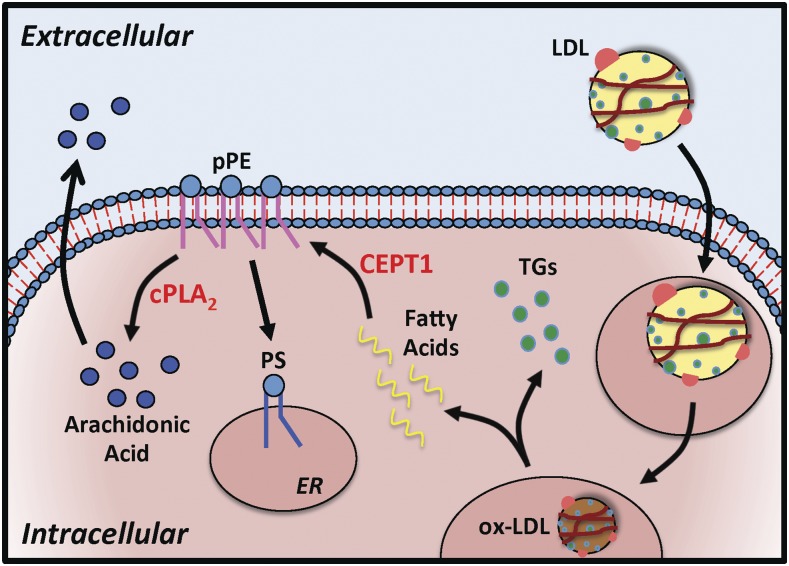
CEPT1-derived pPEs contribute to AA production. LDL particles internalized by vascular macrophages and smooth muscle cells are oxidized (ox-LDL). The ox-LDL is hydrolyzed in the cellular lysosomes and peroxisomes to generate triglycerides (TGs) and free fatty acids. Through the Kennedy pathway, free fatty acids are utilized in the de novo biosynthesis of pPEs. Hydrolysis of pPEs by cPLA_2_ leads to production of AA, which may be released into the extracellular space to alter plaque physiology and progression. pPEs can also be converted to PSs in the ER via PS synthases.

LDL particles internalized by macrophages and vascular smooth muscle cells are oxidized and hydrolyzed into triglycerides and free fatty acids (**Fig. 4**). Through the Kennedy pathway, free fatty acids are utilized in the de novo biosynthesis of plasmalogen phospholipids like pPEs, which affect membrane fluidity and fusion. The *sn*-2 position of pPEs is usually occupied by a polyunsaturated fatty acid, especially AA, which can be released by cPLA_2_ (12). Tissue pPE content is affected by insulin sensitivity and exercise (50, 51), and is significantly altered in conditions such as Alzheimer’s disease (52), Down’s syndrome, schizophrenia (53), and Zellweger syndrome (7, 12). In vascular tissue, this phospholipid class can lead to increased production of advanced glycated end products and AA metabolites, such as eisosanoids and prostaglandins (36). Collectively, this is thought to induce endothelial dysfunction and tissue inflammation, which are strong stimuli for atheroprogression (3, 54). Our data support this model by demonstrating that MIN diseased segments have a higher content of both pPE and AA, with relatively higher AA in both MIN and MAX diseased CEA segments of diabetic subjects (Fig. 4).

In addition to the Kennedy pathway, mammalian cells have an alternative spatially separated pathway that can also facilitate pPE production. At the mitochondrial inner membrane, PS decarboxylase (encoded by *Pisd* gene) can convert PS to PE species with polyunsaturated fatty acids in the *sn*-2 position (55). However, recent gene expression analysis of phospholipid biogenesis enzymes did not find *Pisd* to be differentially expressed in arterial tissue of human diabetic subjects (56). Thus, the contribution of PS decarboxylase in PE and pPE production in human arterial tissue, particularly in the setting of diabetes, remains unclear at this time.

In eukaryotic cells, cPLA_2_ leads to the formation of cellular AA pools via a steady state hydrolysis of the *sn*-2 ester bond of pPEs (33, 57). Consistent with prior findings (32), our data demonstrate differential plaque expression of cPLA_2_, with higher content in both MIN and MAX diseased CEA segments of nondiabetic subjects. No difference was observed in iPLA_2_ expression, suggesting that it does not play as critical of a role in plaque disease severity, and is not influenced by diabetic status. Interestingly, CEPT1 and cPLA_2_ demonstrated opposite expression patterns, suggesting possible negative feedback signaling between the two enzymatic pathways. This is consistent with recent reports demonstrating that CEPT1 substrates, such as cystidine-diphosphocholine and cytidine-diphosphoethanolamine (58, 59), are potent cPLA_2_ inhibitors.

We also observed that specific PS species were altered by diabetic status and that overall there was no significant difference in PCs. This is consistent with prior work that demonstrates no significant relative change in PCs in diabetic arterial specimens (2). However, other reports demonstrate altered PS membrane content in the setting of chronic hyperglycemia and diabetes (43, 60, 61). In the setting of diabetes, increased membrane PSs in red blood cells and endothelial cells are thought to increase cell-cell adhesion and promote vessel thrombosis (60). PS synthases in the ER can covert PC, PE, and pPE into PS (8, 9, 58, 62). Although we did not observe a correlation between the content of pPE and PS in CEA plaque, it is possible that elevated pPE content also contributed to elevated PS content in MIN diseased CEA plaques (Fig. 4). Consistent with prior findings (8, 60, 61), elevated PS content in diabetic CEA plaques is likely an indicator of progressive disease.

In conclusion, our data demonstrate that diabetic subjects with high-grade carotid artery atherosclerosis have increased CEPT1 expression and pPE content in the MIN diseased segments of the carotid plaque. In MIN diseased CEA segments, AA content is also increased relative to MAX diseased segments. These findings provide the first evidence that CEPT1 likely plays an important role in human plaque vulnerability in diabetic subjects. Future investigations targeting CEPT1 and its downstream phospholipid ligands may provide new opportunities for the treatment of human atheroprogression in the setting of diabetes.

## Supplementary Material

Supplemental Data

## References

[1] Falk E. 2006 Pathogenesis of atherosclerosis. J. Am. Coll. Cardiol. 47: C7–C12. 16631513 10.1016/j.jacc.2005.09.068

[2] Edsfeldt A., Duner P., Stahlman M., Mollet I. G., Asciutto G., Grufman H., Nitulescu M., Persson A. F., Fisher R. M., Melander O., 2016 Sphingolipids contribute to human atherosclerotic plaque inflammation. Arterioscler. Thromb. Vasc. Biol. 36: 1132–1140. 27055903 10.1161/ATVBAHA.116.305675

[3] Miyazawa T., Nakagawa K., Shimasaki S., and Nagai R. 2012 Lipid glycation and protein glycation in diabetes and atherosclerosis. Amino Acids. 42: 1163–1170. 20957396 10.1007/s00726-010-0772-3

[4] Nakagawa K., Oak J. H., Higuchi O., Tsuzuki T., Oikawa S., Otani H., Mune M., Cai H., and Miyazawa T. 2005 Ion-trap tandem mass spectrometric analysis of Amadori-glycated phosphatidylethanolamine in human plasma with or without diabetes. J. Lipid Res. 46: 2514–2524. 16150834 10.1194/jlr.D500025-JLR200

[5] Demirkan A., van Duijn C. M., Ugocsai P., Isaacs A., Pramstaller P. P., Liebisch G., Wilson J. F., Johansson A., Rudan I., Aulchenko Y. S., 2012 Genome-wide association study identifies novel loci associated with circulating phospho- and sphingolipid concentrations. PLoS Genet. 8: e1002490. 22359512 10.1371/journal.pgen.1002490 PMC3280968

[6] Annibal A., Riemer T., Jovanovic O., Westphal D., Griesser E., Pohl E. E., Schiller J., Hoffmann R., and Fedorova M. 2016 Structural, biological and biophysical properties of glycated and glycoxidized phosphatidylethanolamines. Free Radic. Biol. Med. 95: 293–307. 27012418 10.1016/j.freeradbiomed.2016.03.011 PMC5937679

[7] Braverman N. E., and Moser A. B. 2012 Functions of plasmalogen lipids in health and disease. Biochim. Biophys. Acta. 1822: 1442–1452. 22627108 10.1016/j.bbadis.2012.05.008

[8] Vance J. E. 2008 Phosphatidylserine and phosphatidylethanolamine in mammalian cells: two metabolically related aminophospholipids. J. Lipid Res. 49: 1377–1387. 18204094 10.1194/jlr.R700020-JLR200

[9] Gibellini F., and Smith T. K. 2010 The Kennedy pathway–de novo synthesis of phosphatidylethanolamine and phosphatidylcholine. IUBMB Life. 62: 414–428. 20503434 10.1002/iub.337

[10] Kennedy E. P., and Weiss S. B. 1956 The function of cytidine coenzymes in the biosynthesis of phospholipides. J. Biol. Chem. 222: 193–214. 13366993

[11] Henneberry A. L., Wistow G., and McMaster C. R. 2000 Cloning, genomic organization, and characterization of a human cholinephosphotransferase. J. Biol. Chem. 275: 29808–29815. 10893425 10.1074/jbc.M005786200

[12] Farooqui A. A., Yang H. C., and Horrocks L. A. 1995 Plasmalogens, phospholipases A2 and signal transduction. Brain Res. Brain Res. Rev. 21: 152–161. 8866672 10.1016/0165-0173(95)00008-9

[13] Zieseniss S., Zahler S., Muller I., Hermetter A., and Engelmann B. 2001 Modified phosphatidylethanolamine as the active component of oxidized low density lipoprotein promoting platelet prothrombinase activity. J. Biol. Chem. 276: 19828–19835. 11278348 10.1074/jbc.M007506200

[14] Ivanova P. T., Milne S. B., and Brown H. A. 2010 Identification of atypical ether-linked glycerophospholipid species in macrophages by mass spectrometry. J. Lipid Res. 51: 1581–1590. 19965583 10.1194/jlr.D003715 PMC3035522

[15] Gregg E. W., Li Y., Wang J., Burrows N. R., Ali M. K., Rolka D., Williams D. E., and Geiss L. 2014 Changes in diabetes-related complications in the United States, 1990–2010. N. Engl. J. Med. 370: 1514–1523. 24738668 10.1056/NEJMoa1310799

[16] Ujihara N., Sakka Y., Takeda M., Hirayama M., Ishii A., Tomonaga O., Babazono T., Takahashi C., Yamashita K., and Iwamoto Y. 2002 Association between plasma oxidized low-density lipoprotein and diabetic nephropathy. Diabetes Res. Clin. Pract. 58: 109–114. 12213352 10.1016/s0168-8227(02)00134-1

[17] Huang Y. S., Horrobin D. F., Manku M. S., Mitchell J., and Ryan M. A. 1984 Tissue phospholipid fatty acid composition in the diabetic rat. Lipids. 19: 367–370. 6738315 10.1007/BF02534790

[18] Stary H. C., Chandler A. B., Dinsmore R. E., Fuster V., Glagov S., Insull W. Jr., Rosenfeld M. E., Schwartz C. J., Wagner W. D., and Wissler R. W. 1995 A definition of advanced types of atherosclerotic lesions and a histological classification of atherosclerosis. A report from the Committee on Vascular Lesions of the Council on Arteriosclerosis, American Heart Association. Arterioscler. Thromb. Vasc. Biol. 15: 1512–1531. 7670967 10.1161/01.atv.15.9.1512

[19] Bligh E. G., and Dyer W. J. 1959 A rapid method of total lipid extraction and purification. Can. J. Biochem. Physiol. 37: 911–917. 13671378 10.1139/o59-099

[20] Hsu F-F., and Turk J. 2009 Electrospray ionization with low-energy collisionally activated dissociation tandem mass spectrometry of glycerophospholipids: Mechanisms of fragmentation and structural characterization. J. Chromatogr. B Analyt. Technol. Biomed. Life Sci. 877: 2673–2695. 10.1016/j.jchromb.2009.02.033 PMC2723218 19269264

[21] Hsu F. F., and Turk J. 2003 Electrospray ionization/tandem quadrupole mass spectrometric studies on phosphatidylcholines: the fragmentation processes. J. Am. Soc. Mass Spectrom. 14: 352–363. 12686482 10.1016/S1044-0305(03)00064-3

[22] Hsu F-F. 2016 Complete structural characterization of ceramides as [M-H]- ions by multiple-stage linear ion trap mass spectrometry. Biochimie. 130: 63–75. 27523779 10.1016/j.biochi.2016.07.012 PMC5086283

[23] Hsu F. F., Kuhlmann F. M., Turk J., and Beverley S. M. 2014 Multiple-stage linear ion-trap with high resolution mass spectrometry towards complete structural characterization of phosphatidylethanolamines containing cyclopropane fatty acyl chain in Leishmania infantum. J. Mass Spectrom. 49: 201–209. 24619546 10.1002/jms.3327 PMC4007172

[24] Hsu F. F., and Turk J. 2000 Structural determination of sphingomyelin by tandem mass spectrometry with electrospray ionization. J. Am. Soc. Mass Spectrom. 11: 437–449. 10790848 10.1016/S1044-0305(99)00150-6

[25] Hsu F. F., and Turk J. 2005 Tandem mass spectrometry with electrospray ionization of sphingomyelins. *In* The Encyclopedia of Mass Spectrometry, Vol. III. Applications in Biochemistry, Biology, and Medicine. A. Part, M. L. Gross, and R. Caprioli, editors. Elsevier Science, New York. 430–447.

[26] Hsu F-F., and Turk J. 2000 Charge-driven fragmentation processes in diacyl glycerophosphatidic acids upon low-energy collisional activation. A mechanistic proposal. J. Am. Soc. Mass Spectrom. 11: 797–803. 10976887 10.1016/S1044-0305(00)00151-3

[27] Hsu F-F., and Turk J. 2005 Studies on phosphatidylserine by tandem quadrupole and multiple stage quadrupole ion-trap mass spectrometry with electrospray ionization: Structural characterization and the fragmentation processes. J. Am. Soc. Mass Spectrom. 16: 1510–1522. 16023863 10.1016/j.jasms.2005.04.018

[28] Hsu F-F., Turk J., Zhang K., and Beverley S. 2007 Characterization of inositol phosphorylceramides from Leishmania major by tandem mass spectrometry with electrospray ionization. J. Am. Soc. Mass Spectrom. 18: 1591–1604. 17627842 10.1016/j.jasms.2007.05.017 PMC2065762

[29] Hsu F. F., Kuhlmann F. M., Turk J., and Beverley S. M. 2014 Multiple-stage linear ion-trap with high resolution mass spectrometry towards complete structural characterization of phosphatidylethanolamines containing cyclopropane fatty acyl chain in Leishmania infantum. J. Mass Spectrom. 49: 201–209. 24619546 10.1002/jms.3327 PMC4007172

[30] Carr S., Farb A., Pearce W. H., Virmani R., and Yao J. S. 1996 Atherosclerotic plaque rupture in symptomatic carotid artery stenosis. J. Vasc. Surg. 23: 755–765. 8667496 10.1016/s0741-5214(96)70237-9

[31] Funai K., Lodhi I. J., Spears L. D., Yin L., Song H., Klein S., and Semenkovich C. F. 2016 Skeletal muscle phospholipid metabolism regulates insulin sensitivity and contractile function. Diabetes. 65: 358–370. 26512026 10.2337/db15-0659 PMC4747455

[32] Elinder L. S., Dumitrescu A., Larsson P., Hedin U., Frostegard J., and Claesson H. E. 1997 Expression of phospholipase A2 isoforms in human normal and atherosclerotic arterial wall. Arterioscler. Thromb. Vasc. Biol. 17: 2257–2263. 9351398 10.1161/01.atv.17.10.2257

[33] Balsinde J., Winstead M. V., and Dennis E. A. 2002 Phospholipase A(2) regulation of arachidonic acid mobilization. FEBS Lett. 531: 2–6. 12401193 10.1016/s0014-5793(02)03413-0

[34] Gregor M. F., and Hotamisligil G. S. 2011 Inflammatory mechanisms in obesity. Annu. Rev. Immunol. 29: 415–445. 21219177 10.1146/annurev-immunol-031210-101322

[35] Kanter J. E., Kramer F., Barnhart S., Averill M. M., Vivekanandan-Giri A., Vickery T., Li L. O., Becker L., Yuan W., Chait A., 2012 Diabetes promotes an inflammatory macrophage phenotype and atherosclerosis through acyl-CoA synthetase 1. Proc. Natl. Acad. Sci. USA. 109: E715–E724. 22308341 10.1073/pnas.1111600109 PMC3311324

[36] Watkins S. M., and Hotamisligil G. S. 2012 Promoting atherosclerosis in type 1 diabetes through the selective activation of arachidonic acid and PGE(2) production. Circ. Res. 111: 394–396. 22859668 10.1161/CIRCRESAHA.112.273508

[37] Alhusban A., and Fagan S. C. 2011 Secondary prevention of stroke in the elderly: a review of the evidence. Am. J. Geriatr. Pharmacother. 9: 143–152. 21570361 10.1016/j.amjopharm.2011.04.002

[38] Balk E. M., Karas R. H., Jordan H. S., Kupelnick B., Chew P., and Lau J. 2004 Effects of statins on vascular structure and function: a systematic review. Am. J. Med. 117: 775–790. 15541327 10.1016/j.amjmed.2004.05.026

[39] Brott T. G., Halperin J. L., Abbara S., Bacharach J. M., Barr J. D., Bush R. L., Cates C. U., Creager M. A., Fowler S. B., Friday G., 2011 ASA/ACCF/AHA/AANN/AANS/ACR/ASNR/CNS/SAIP/SCAI/SIR/SNIS/SVM/SVS guideline on the management of patients with extracranial carotid and vertebral artery disease: executive summary. A report of the American College of Cardiology Foundation/American Heart Association Task Force on Practice Guidelines, and the American Stroke Association, American Association of Neuroscience Nurses, American Association of Neurological Surgeons, American College of Radiology, American Society of Neuroradiology, Congress of Neurological Surgeons, Society of Atherosclerosis Imaging and Prevention, Society for Cardiovascular Angiography and Interventions, Society of Interventional Radiology, Society of NeuroInterventional Surgery, Society for Vascular Medicine, and Society for Vascular Surgery. Circulation. 124: 489–532. [Erratum. 2011. *Circulation*. 124: e145.] 21282505 10.1161/CIR.0b013e31820d8d78

[40] Jellinger P. S., Handelsman Y., Rosenblit P. D., Bloomgarden Z. T., Fonseca V. A., Garber A. J., Grunberger G., Guerin C. K., Bell D. S. H., Mechanick J. I., 2017 American Association of Clinical Endocrinologists and American College of Endocrinology guidelines for management of dyslipidemia and prevention of cardiovascular disease. Endocr. Pract. 23: 1–87. 10.4158/EP171764.APPGL 28437620

[41] Spanos K., Petrocheilou G., Karathanos C., Labropoulos N., Mikhailidis D., and Giannoukas A. 2017 Carotid bifurcation geometry and atherosclerosis. Angiology. 68: 757–764. 28164715 10.1177/0003319716678741

[42] Ménégaut L., Masson D., Abello N., Denimal D., Truntzer C., Ducoroy P., Lagrost L., Pais de Barros J. P., Athias A., Petit J. M., 2016 Specific enrichment of 2-arachidonoyl-lysophosphatidylcholine in carotid atheroma plaque from type 2 diabetic patients. Atherosclerosis. 251: 339–347. 27189320 10.1016/j.atherosclerosis.2016.05.004

[43] Vance J. E., and Tasseva G. 2013 Formation and function of phosphatidylserine and phosphatidylethanolamine in mammalian cells. Biochim. Biophys. Acta. 1831: 543–554. 22960354 10.1016/j.bbalip.2012.08.016

[44] Saybolt M. D., Lilly S. M., Patel D., Hamamdzic D., Llano R., Fenning R. S., Madden S., and Wilensky R. L. 2016 The vulnerable artery: early and rapid deposition of lipid in coronary arteries is associated with subsequent development of thin-cap fibroatheromas. EuroIntervention. 11: e1612–e1618. 27056122 10.4244/EIJV11I14A312

[45] Janoudi A., Shamoun F. E., Kalavakunta J. K., and Abela G. S. 2016 Cholesterol crystal induced arterial inflammation and destabilization of atherosclerotic plaque. Eur. Heart J. 37: 1959–1967. 26705388 10.1093/eurheartj/ehv653

[46] Bentzon J. F., Otsuka F., Virmani R., and Falk E. 2014 Mechanisms of plaque formation and rupture. Circ. Res. 114: 1852–1866. 24902970 10.1161/CIRCRESAHA.114.302721

[47] Maldonado N., Kelly-Arnold A., Vengrenyuk Y., Laudier D., Fallon J. T., Virmani R., Cardoso L., and Weinbaum S. 2012 A mechanistic analysis of the role of microcalcifications in atherosclerotic plaque stability: potential implications for plaque rupture. Am. J. Physiol. Heart Circ. Physiol. 303: H619–H628. 22777419 10.1152/ajpheart.00036.2012 PMC3468470

[48] Chakravarthy M. V., Lodhi I. J., Yin L., Malapaka R. R., Xu H. E., Turk J., and Semenkovich C. F. 2009 Identification of a physiologically relevant endogenous ligand for PPARalpha in liver. Cell. 138: 476–488. 19646743 10.1016/j.cell.2009.05.036 PMC2725194

[49] Funai K., and Semenkovich C. F. 2011 Skeletal muscle lipid flux: running water carries no poison. Am. J. Physiol. Endocrinol. Metab. 301: E245–E251. 21558546 10.1152/ajpendo.00152.2011 PMC3275151

[50] Newsom S. A., Brozinick J. T., Kiseljak-Vassiliades K., Strauss A. N., Bacon S. D., Kerege A. A., Bui H. H., Sanders P., Siddall P., Wei T., 2016 Skeletal muscle phosphatidylcholine and phosphatidylethanolamine are related to insulin sensitivity and respond to acute exercise in humans. J. Appl. Physiol. (1985). 120: 1355–1363. 27032901 10.1152/japplphysiol.00664.2015 PMC4891931

[51] van der Veen J. N., Lingrell S., da Silva R. P., Jacobs R. L., and Vance D. E. 2014 The concentration of phosphatidylethanolamine in mitochondria can modulate ATP production and glucose metabolism in mice. Diabetes. 63: 2620–2630. 24677714 10.2337/db13-0993

[52] Smith M. A., Taneda S., Richey P. L., Miyata S., Yan S. D., Stern D., Sayre L. M., Monnier V. M., and Perry G. 1994 Advanced Maillard reaction end products are associated with Alzheimer disease pathology. Proc. Natl. Acad. Sci. USA. 91: 5710–5714. 8202552 10.1073/pnas.91.12.5710 PMC44066

[53] Kaddurah-Daouk R., Mc J., Evoy R., Baillie H., Zhu K. Y. J., Nimgaonkar V. L., Buckley P. F., Keshavan M. S., Georgiades A., and Nasrallah H. A. 2012 Impaired plasmalogens in patients with schizophrenia. Psychiatry Res. 198: 347–352. 22513041 10.1016/j.psychres.2012.02.019

[54] Kume S., Takeya M., Mori T., Araki N., Suzuki H., Horiuchi S., Kodama T., Miyauchi Y., and Takahashi K. 1995 Immunohistochemical and ultrastructural detection of advanced glycation end products in atherosclerotic lesions of human aorta with a novel specific monoclonal antibody. Am. J. Pathol. 147: 654–667. 7545874 PMC1870970

[55] Bleijerveld O. B., Brouwers J. F., Vaandrager A. B., Helms J. B., and Houweling M. 2007 The CDP-ethanolamine pathway and phosphatidylserine decarboxylation generate different phosphatidylethanolamine molecular species. J. Biol. Chem. 282: 28362–28372. 17673461 10.1074/jbc.M703786200

[56] Skov V., Knudsen S., Olesen M., Hansen M. L., and Rasmussen L. M. 2012 Global gene expression profiling displays a network of dysregulated genes in non-atherosclerotic arterial tissue from patients with type 2 diabetes. Cardiovasc. Diabetol. 11: 15. 22340758 10.1186/1475-2840-11-15 PMC3348024

[57] Gijón M. A., and Leslie C. C. 1999 Regulation of arachidonic acid release and cytosolic phospholipase A2 activation. J. Leukoc. Biol. 65: 330–336. 10080535 10.1002/jlb.65.3.330

[58] Fagone P., and Jackowski S. 2013 Phosphatidylcholine and the CDP-choline cycle. Biochim. Biophys. Acta. 1831: 523–532. 23010477 10.1016/j.bbalip.2012.09.009 PMC3562404

[59] Bleijerveld O. B., Klein W., Vaandrager A. B., Helms J. B., and Houweling M. 2004 Control of the CDPethanolamine pathway in mammalian cells: effect of CTP:phosphoethanolamine cytidylyltransferase overexpression and the amount of intracellular diacylglycerol. Biochem. J. 379: 711–719. 14759225 10.1042/BJ20031422 PMC1224125

[60] Wautier M. P., Heron E., Picot J., Colin Y., Hermine O., and Wautier J. L. 2011 Red blood cell phosphatidylserine exposure is responsible for increased erythrocyte adhesion to endothelium in central retinal vein occlusion. J. Thromb. Haemost. 9: 1049–1055. 21362128 10.1111/j.1538-7836.2011.04251.x

[61] Zwaal R. F., Comfurius P., and Bevers E. M. 2005 Surface exposure of phosphatidylserine in pathological cells. Cell. Mol. Life Sci. 62: 971–988. 15761668 10.1007/s00018-005-4527-3 PMC11924510

[62] Hishikawa D., Hashidate T., Shimizu T., and Shindou H. 2014 Diversity and function of membrane glycerophospholipids generated by the remodeling pathway in mammalian cells. J. Lipid Res. 55: 799–807. 24646950 10.1194/jlr.R046094 PMC3995458

