# Supplementary material for: Diabetes adversely affects phospholipid profiles in human carotid artery endarterectomy plaques
Source: J Lipid Res. 2018 Feb 24;59(4):730–8. doi: 10.1194/jlr.M081026 (PMC5880490; doi:10.1194/jlr.M081026)
Supplement: Supplemental Data [file supp_59_4_730__index.html]

Diabetes Adversely Affects Phospholipid Profiles in Human Carotid Artery Endarterectomy Plaques — Diabetes adversely affects phospholipid profiles in human carotid artery endarterectomy plaques — Supplemental Data 

# Diabetes adversely affects phospholipid profiles in human carotid artery endarterectomy plaques

## Supplemental Data

- Supplemental Tables and Figures (.pdf, 1.4 MB) - Supplemental Tables and Figures
