# Supplementary material for: Diabetes adversely affects phospholipid profiles in human carotid artery endarterectomy plaques
Source: J Lipid Res. 2018 Feb 24;59(4):730–8. doi: 10.1194/jlr.M081026 (PMC5880490; doi:10.1194/jlr.M081026)
Supplement: Supplemental Data [file 10.1194_M081026_jlr.M081026-1.pdf]

Supplemental Tables:

Supplemental Table S1: Demographics of patients used for CEPT1 Western blot analysis and mass spectrometry lipidomic analysis.

	Non-Diabetic (n = 7)	Diabetic (n = 10)	P Value
**Age**			
**50-60 (%)**	14	10	0.80
**61-70 (%)**	29	30	0.95
**71-80 (%)**	57	50	0.79
**80-90 (%)**	10	0	0.42
**Demographics**			
**Gender (#)**	M5/F2	M6/F4	0.65
**BMI ≥ 30 (%)**	14	60	0.07
**Current Smoker (%)**	14	30	0.48
**Hypertension (%)**	57	90	0.13
**Hyperlipidemia (%)**	71	90	0.35
**CAD (%)**	14	50	0.48
**Medications**			
**Antiplatelet (%)**	100	80	0.23
**Beta-Blocker (%)**	14	80	0.005*
**Statin (%)**	71	100	0.08
**Insulin (%)**	0	30	<0.01*

** Significance with a non-parametric Mann-Whitney U test.*

Supplemental Table S2: Summary of phosphatidylcholine (PC) species evaluated in CEA specimens using electrospray ionization mass spectrometry

Species	Formula	Most Abundant Mass
PC(14:0/14:0)(IS)	C 36 H 73 O 8 N 1 P 1	678.5
PC30:0	C 38 H 77 O 8 N 1 P 1	706.5
PC30:1	C 38 H 75 O 8 N 1 P 1	704.5
PC30:2	C 38 H 73 O 8 N 1 P 1	702.5
PC32:0	C 40 H 81 O 8 N 1 P 1	734.6
PC32:1	C 40 H 79 O 8 N 1 P 1	732.6
PC32:2	C 40 H 77 O 8 N 1 P 1	730.5
PC34:0	C 42 H 85 O 8 N 1 P 1	762.6
PC34:1*	C 42 H 83 O 8 N 1 P 1	760.6
PC34:2	C 42 H 81 O 8 N 1 P 1	758.6
PC34:3	C 42 H 79 O 8 N 1 P 1	756.6
PC36:1	C 44 H 87 O 8 N 1 P 1	788.6
PC36:2	C 44 H 85 O 8 N 1 P 1	786.6
PC36:3	C 44 H 83 O 8 N 1 P 1	784.6
PC36:4	C 44 H 81 O 8 N 1 P 1	782.6
PC36:5	C 44 H 79 O 8 N 1 P 1	780.6
PC38:2	C 46 H 89 O 8 N 1 P 1	814.6
PC38:3	C 46 H 87 O 8 N 1 P 1	812.6
PC38:4	C 46 H 85 O 8 N 1 P 1	810.6
PC38:5	C 46 H 83 O 8 N 1 P 1	808.6
PC40:3	C 48 H 91 O 8 N 1 P 1	840.6
PC40:4	C 48 H 89 O 8 N 1 P 1	838.6
PC40:5	C 48 H 87 O 8 N 1 P 1	836.6
PC40:6	C 48 H 85 O 8 N 1 P 1	834.6

IS, internal standard
* most abundant phospholipid

Supplemental Table S3: Summary of alkyl ether phosphatidylcholine (aPC) species evaluated in CEA specimens using electrospray ionization mass spectrometry

Species	Formula	Most Abundant Mass
PC(14:0/14:0)(IS)	C 36 H 73 O 8 N 1 P 1	678.5
aPC30:0	C 38 H 79 O 7 N 1 P 1	692.6
aPC30:1	C 38 H 77 O 7 N 1 P 1	690.5
aPC32:0	C 40 H 83 O 7 N 1 P 1	720.6
aPC32:1	C 40 H 81 O 7 N 1 P 1	718.6
aPC32:2	C 40 H 79 O 7 N 1 P 1	716.6
aPC34:0	C 42 H 87 O 7 N 1 P 1	748.6
aPC34:1*	C 42 H 85 O 7 N 1 P 1	746.6
aPC34:2	C 42 H 83 O 7 N 1 P 1	744.6
aPC36:1	C 44 H 89 O 7 N 1 P 1	774.6
aPC36:2	C 44 H 87 O 7 N 1 P 1	772.6
aPC36:3	C 44 H 85 O 7 N 1 P 1	770.6
aPC36:4	C 44 H 83 O 7 N 1 P 1	768.6
aPC38:2	C 46 H 91 O 7 N 1 P 1	800.7
aPC38:3	C 46 H 89 O 7 N 1 P 1	798.6
aPC38:4	C 46 H 87 O 7 N 1 P 1	796.6
aPC38:5	C 46 H 85 O 7 N 1 P 1	794.6

IS, internal standard
* most abundant phospholipid

Supplemental Table S4: Summary of sphingomyelin (SPM) species evaluated in CEA specimens using electrospray ionization mass spectrometry

Species	Formula	Most Abundant Mass
PC(14:0/14:0)(IS)	C 36 H 73 O 8 N 1 P 1	678.5
SPM34:1*	C 39 H 80 N 2 O 6 P 1	703.6
SPM34:2	C 39 H 78 N 2 O 6 P 1	701.6
SPM36:1	C 41 H 84 N 2 O 6 P 1	731.6
SPM36:2	C 41 H 82 N 2 O 6 P 1	729.6
SPM38:1	C 43 H 88 N 2 O 6 P 1	759.6
SPM39:1	C 44 H 90 N 2 O 6 P 1	773.7
SPM40:1	C 45 H 92 N 2 O 6 P 1	787.7
SPM41:1	C 46 H 94 N 2 O 6 P 1	801.7
SPM42:1	C 47 H 96 N 2 O 6 P 1	815.7
SPM42:2	C 47 H 94 N 2 O 6 P 1	813.7
SPM42:3	C 47 H 92 N 2 O 6 P 1	811.7

IS, internal standard
* most abundant phospholipid

Supplemental Table S5: Summary of phosphatidylserine (PS) species evaluated in CEA specimens using electrospray ionization mass spectrometry

Species	Formula	Most Abundant Mass
PS(14:0/14:0)(IS)	C 34 H 65 O 10 N 1 P 1	678.4
PS32:0	C 38 H 73 O 10 N 1 P 1	734.5
PS32:1	C 38 H 71 O 10 N 1 P 1	732.5
PS34:1	C 40 H 75 O 10 N 1 P 1	760.5
PS34:2	C 40 H 73 O 10 N 1 P 1	758.5
PS36:0	C 42 H 81 O 10 N 1 P 1	790.6
PS36:1*	C 42 H 79 O 10 N 1 P 1	788.5
PS36:2	C 42 H 77 O 10 N 1 P 1	786.5
PS36:3	C 42 H 75 O 10 N 1 P 1	784.5
PS36:4	C 42 H 73 O 10 N 1 P 1	782.5
PS38:1	C 44 H 83 O 10 N 1 P 1	816.6
PS38:2	C 44 H 81 O 10 N 1 P 1	814.6
PS38:3	C 44 H 79 O 10 N 1 P 1	812.5
PS38:4	C 44 H 77 O 10 N 1 P 1	810.5
PS38:5	C 44 H 75 O 10 N 1 P 1	808.5
PS40:1	C 46 H 87 O 10 N 1 P 1	844.6
PS40:2	C 46 H 85 O 10 N 1 P 1	842.6
PS40:3	C 46 H 83 O 10 N 1 P 1	840.6
PS40:4	C 46 H 81 O 10 N 1 P 1	838.6
PS40:5	C 46 H 79 O 10 N 1 P 1	836.5
PS40:6	C 46 H 77 O 10 N 1 P 1	834.5
PS48:2	C 48 H 93 O 10 N 1 P 1	874.7
PS48:3	C 48 H 91 O 10 N 1 P 1	872.6
PS48:4	C 48 H 89 O 10 N 1 P 1	870.6
PS48:5	C 48 H 87 O 10 N 1 P 1	868.6
PS48:6	C 48 H 85 O 10 N 1 P 1	866.6

IS, internal standard
* most abundant phospholipid

Supplemental Table S6: Summary of phosphatidylethanolamine (PE) species evaluated in CEA specimens using electrospray ionization mass spectrometry

Species	Formula	Most Abundant Mass
PE(14:0/14:0)(IS)	C 33 H 65 N 1 O 8 P 1	634.4
PE34:1	C 39 H 75 N 1 O 8 P 1	716.5
PE34:2	C 39 H 73 N 1 O 8 P 1	714.5
PE36:2	C 41 H 77 N 1 O 8 P 1	742.5
PE36:3	C 41 H 75 N 1 O 8 P 1	740.5
PE36:4	C 41 H 73 N 1 O 8 P 1	738.5
PE38:4*	C 43 H 77 N 1 O 8 P 1	766.5
PE38:5	C 43 H 75 N 1 O 8 P 1	764.5
PE38:6	C 43 H 73 N 1 O 8 P 1	762.5
PE40:5	C 45 H 79 N 1 O 8 P 1	792.6
PE40:6	C 45 H 77 N 1 O 8 P 1	790.5
PE40:7	C 45 H 75 N 1 O 8 P 1	788.5

IS, internal standard
* most abundant phospholipid

Supplemental Table S7: Summary of plasmenyl-phosphatidylethanolamine (pPE) species evaluated in CEA specimens using electrospray ionization mass spectrometry

Species	Formula	Most Abundant Mass
PE(14:0/14:0)(IS)	C 33 H 65 N 1 O 8 P 1	634.4
pPE34:1	C 39 H 75 N 1 O 7 P 1	700.5
pPE34:2	C 39 H 73 N 1 O 7 P 1	698.5
pPE36:2	C 41 H 77 N 1 O 7 P 1	726.5
pPE36:3	C 41 H 75 N 1 O 7 P 1	724.5
pPE36:4	C 41 H 73 N 1 O 7 P 1	722.5
pPE38:4*	C 43 H 77 N 1 O 7 P 1	750.5
pPE38:5	C 43 H 75 N 1 O 7 P 1	748.5
pPE38:6	C 43 H 73 N 1 O 7 P 1	746.5
pPE40:5	C 45 H 79 N 1 O 7 P 1	776.6
pPE40:6	C 45 H 77 N 1 O 7 P 1	774.5
pPE40:7	C 45 H 75 N 1 O 7 P 1	772.5

IS, internal standard
* most abundant phospholipid

Supplemental Table S8: Summary of phosphatidylglycerol (PG) species evaluated in CEA specimens using electrospray ionization mass spectrometry

Species	Formula	Most Abundant Mass
PG(14:0/14:0)(IS)	C 34 H 66 O 10 P 1	665.4
PG30:0*	C 36 H 70 O 10 P 1	693.5
PG32:1	C 38 H 72 O 10 P 1	719.5
PG32:0	C 38 H 74 O 10 P 1	721.5
PG34:2	C 40 H 74 O 10 P 1	745.5
PG34:1	C 40 H 76 O 10 P 1	747.5
PG34:0	C 40 H 78 O 10 P 1	749.5
PG36:4	C 42 H 74 O 10 P 1	769.5
PG36:3	C 42 H 76 O 10 P 1	771.5
PG36:2	C 42 H 78 O 10 P 1	773.5
PG36:1	C 42 H 80 O 10 P 1	775.5
PG38:4	C 44 H 78 O 10 P 1	797.5
PG38:3	C 44 H 80 O 10 P 1	799.5
PG38:2	C 44 H 82 O 10 P 1	801.6

IS, internal standard
* most abundant phospholipid

Supplemental Table S9: Summary of phosphatidylinositol (PI) species evaluated in CEA specimens using electrospray ionization mass spectrometry

Species	Formula	Most Abundant Mass
PI(16:0/16:0)(IS)	C 41 H 78 O 13 P 1	809.5
PI32:1	C 41 H 76 O 13 P 1	807.5
PI34:0	C 43 H 82 O 13 P 1	837.5
PI34:1	C 43 H 80 O 13 P 1	835.5
PI34:2	C 43 H 78 O 13 P 1	833.5
PI36:0	C 45 H 86 O 13 P 1	865.6
PI36:1	C 45 H 84 O 13 P 1	863.6
PI36:2	C 45 H 82 O 13 P 1	861.5
PI36:3	C 45 H 80 O 13 P 1	859.5
PI36:4	C 45 H 78 O 13 P 1	857.5
PI38:2	C 47 H 86 O 13 P 1	889.6
PI38:3	C 47 H 84 O 13 P 1	887.6
PI38:4*	C 47 H 82 O 13 P 1	885.5
PI38:5	C 47 H 80 O 13 P 1	883.5
PI40:3	C 49 H 88 O 13 P 1	915.6
PI40:4	C 49 H 86 O 13 P 1	913.6
PI40:5	C 49 H 84 O 13 P 1	911.6

IS, internal standard
* most abundant phospholipid

Supplemental Table S10: Summary of ceramide (Cer) species evaluated in CEA specimens using electrospray ionization mass spectrometry

Species	Formula	Most Abundant Mass
Cer(d18:1/12:0)(IS)	C 30 H 58 N 1 O 3	480.4
Cer(d18:1/16:0)*	C 34 H 66 N 1 O 3	536.5
Cer(d18:1/18:0)	C 36 H 70 N 1 O 3	564.5
Cer(d18:1/20:0)	C 38 H 74 N 1 O 3	592.6
Cer(d18:1/22:0)	C 40 H 78 N 1 O 3	620.6
Cer(d18:1/23:0)	C 41 H 80 N 1 O 3	634.6
Cer(d18:1/24:0)	C 42 H 82 N 1 O 3	648.6
Cer(d18:1/24:1)	C 42 H 80 N 1 O 3	646.6

IS, internal standard
* most abundant phospholipid

Supplemental Table S11: Demographics of patients used for $cPLA_2$ and $iPLA_2$ Western blot analysis, and AA ELISA.

	Non-Diabetic (n = 5)	Diabetic (n = 7)	P Value
**Age**			
**50-60 (%)**	20	0	0.42
**61-70 (%)**	0	57	0.08
**71-80 (%)**	60	29	0.56
**80-90 (%)**	20	14	0.99
**Demographics**			
**Gender (#)**	M5/F0	M6/F1	0.42
**BMI ≥ 30 (%)**	0	57	0.04*
**Current Smoker (%)**	0	14	0.99
**Hypertension (%)**	100	100	0.99
**Hyperlipidemia (%)**	80	86	0.99
**CAD (%)**	60	71	0.99
**Medications**			
**Antiplatelet (%)**	100	57	0.20
**Beta-Blocker (%)**	20	14	0.99
**Statin (%)**	40	86	0.22
**Insulin (%)**	80	57	0.58

Supplemental Table S12: Demographics of patients used for *cept1* RT-PCR

	Non-Diabetic (n = 15)	Diabetic (n = 8)	P Value
**Age**			
**50-60 (%)**	20	13	0.99
**61-70 (%)**	40	50	0.69
**71-80 (%)**	27	25	0.99
**80-90 (%)**	13	13	0.99
**Demographics**			
**Gender (#)**	M10/F5	M4/F4	0.49
**BMI ≥ 30 (%)**	13	63	0.01*
**Current Smoker (%)**	13	25	0.59
**Hypertension (%)**	80	100	0.29
**Hyperlipidemia (%)**	87	88	0.99
**CAD (%)**	47	50	0.99
**Medications**			
**Antiplatelet (%)**	100	88	0.56
**Beta-Blocker (%)**	20	63	0.07
**Statin (%)**	87	88	0.99
**Insulin (%)**	0	38	<0.01*

** Significance with a non-parametric Mann-Whitney U test.*

Supplemental Table S13: Paired phospholipid analysis between MAX and MIN diseased CEA segments relative to symptomatic versus asymptomatic carotid artery stenosis.

	Symptomatic			Asymptomatic		
**Lipid Family**	**MAX (nmol/ug)**	**MIN (nmol/ug)**	**P value**	**MAX (nmol/ug)**	**MIN (nmol/ug)**	**P value**
PC	0.61	0.73	0.38	0.65	0.76	0.49
aPC	0.28	0.31	0.81	0.31	0.31	1
SPM	1.41	1.59	0.69	1.58	1.56	0.92
PE	0.23	0.24	0.38	0.23	0.24	0.92
pPE	0.29	0.42	0.03 *	0.3	0.51	0.03 *
PI	0.14	0.17	0.3	0.14	0.21	0.11
Cer	0.05	0.04	0.58	0.06	0.05	0.77
PS	0.19	0.39	0.02 *	0.19	0.49	0.02 *
PG	0.02	0.02	0.58	0.02	0.02	0.76

* *Significance with a univariate signed-rank test.*

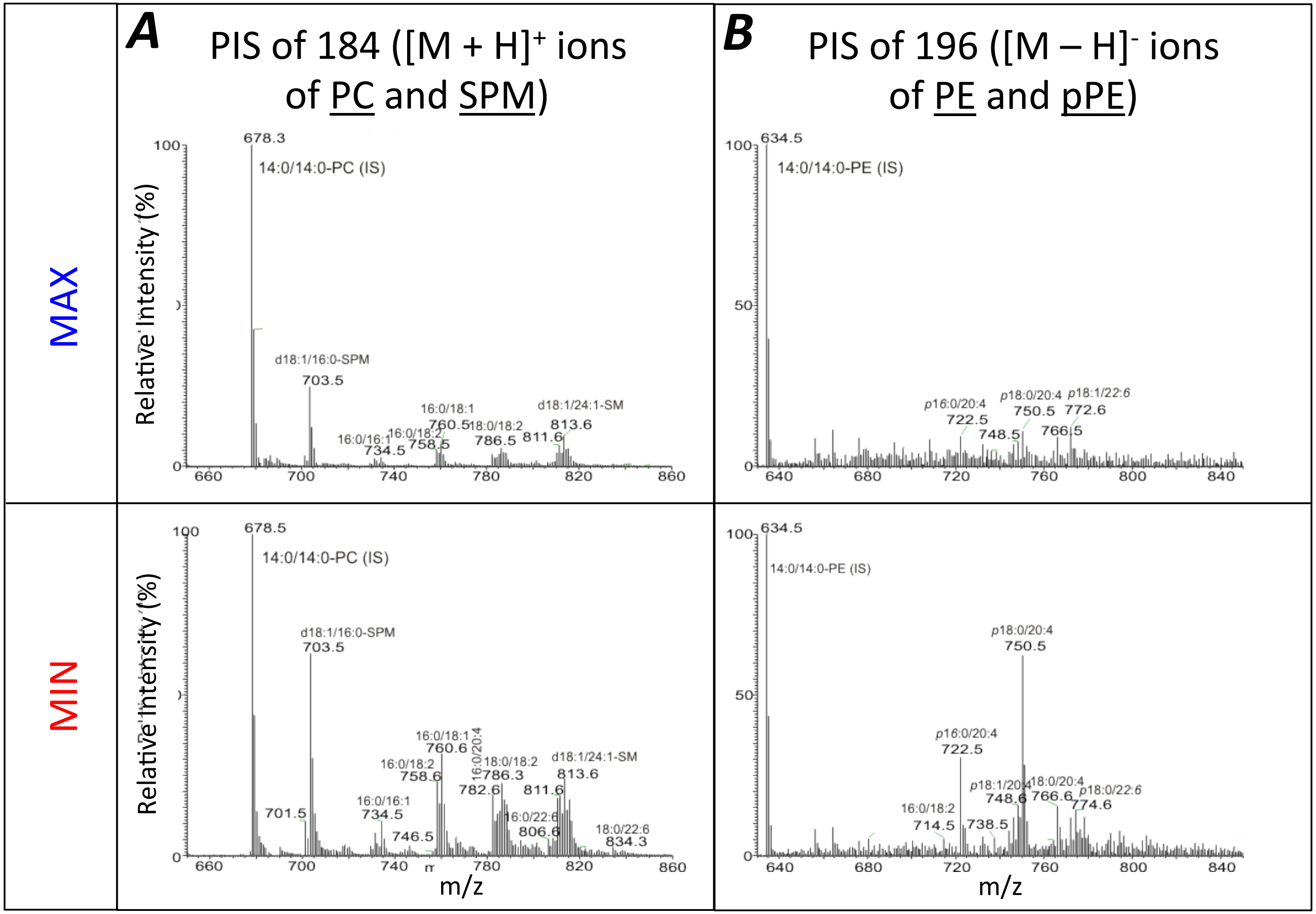

Supplemental Figure S1: PC, SPM, PE, and pPE linked tandem mass spectra scans of representative lipid extracts of MAX and MIN diseased CEA segments of a non-diabetic subject. A) Mass spectra demonstrating the relative intensity of PC and SPM lipid species. B) Mass spectra demonstrating the relative intensity of PE and pPE lipid species. IS, Internal Standard.

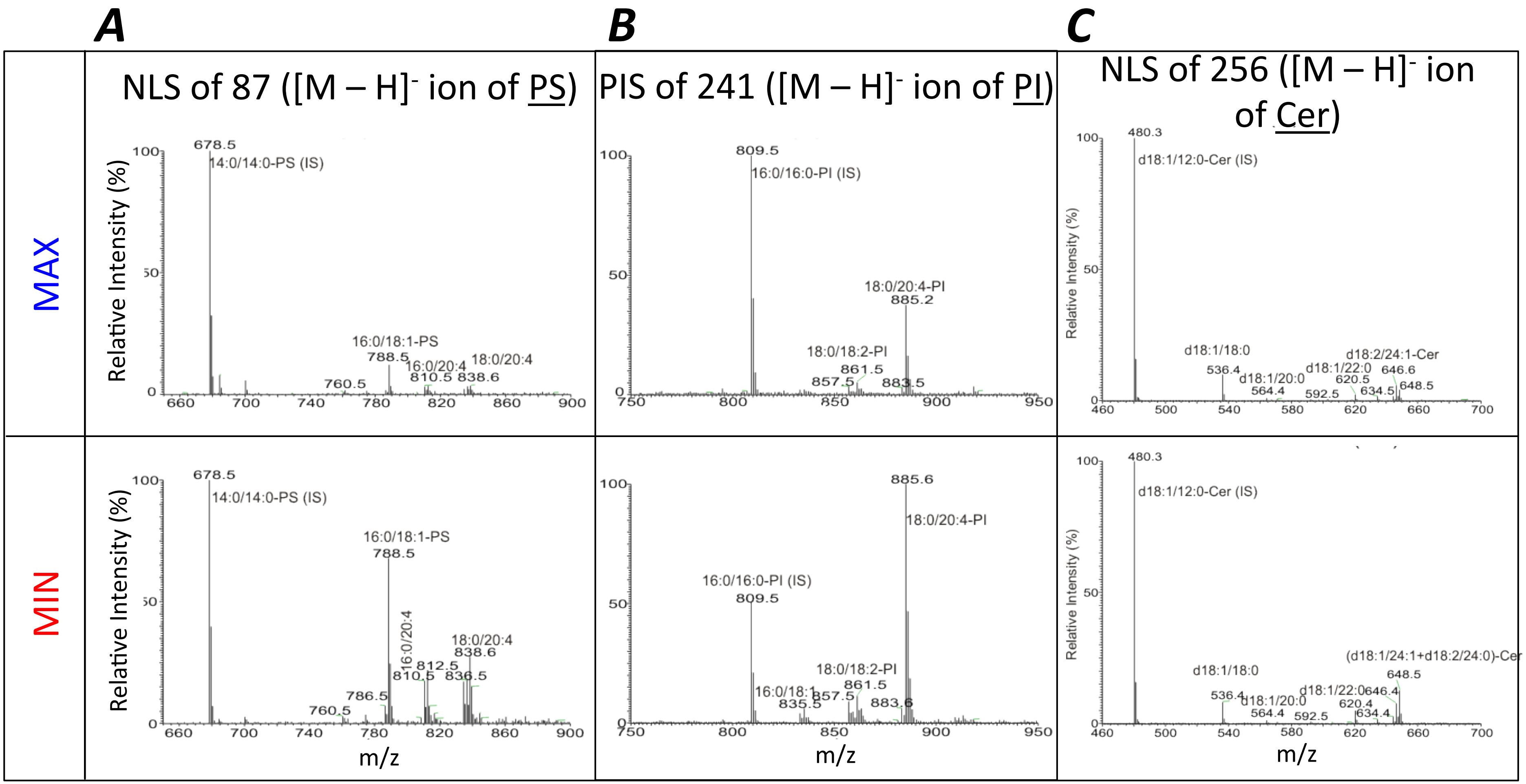

Supplemental Figure S2: PS, PI, and Cer linked tandem mass spectra scans of representative lipid extracts of MAX and MIN diseased CEA segments of a non-diabetic subject. Mass spectra demonstrating the relative intensity of PS (A), PI (B), and Cer (C). IS, Internal Standard.

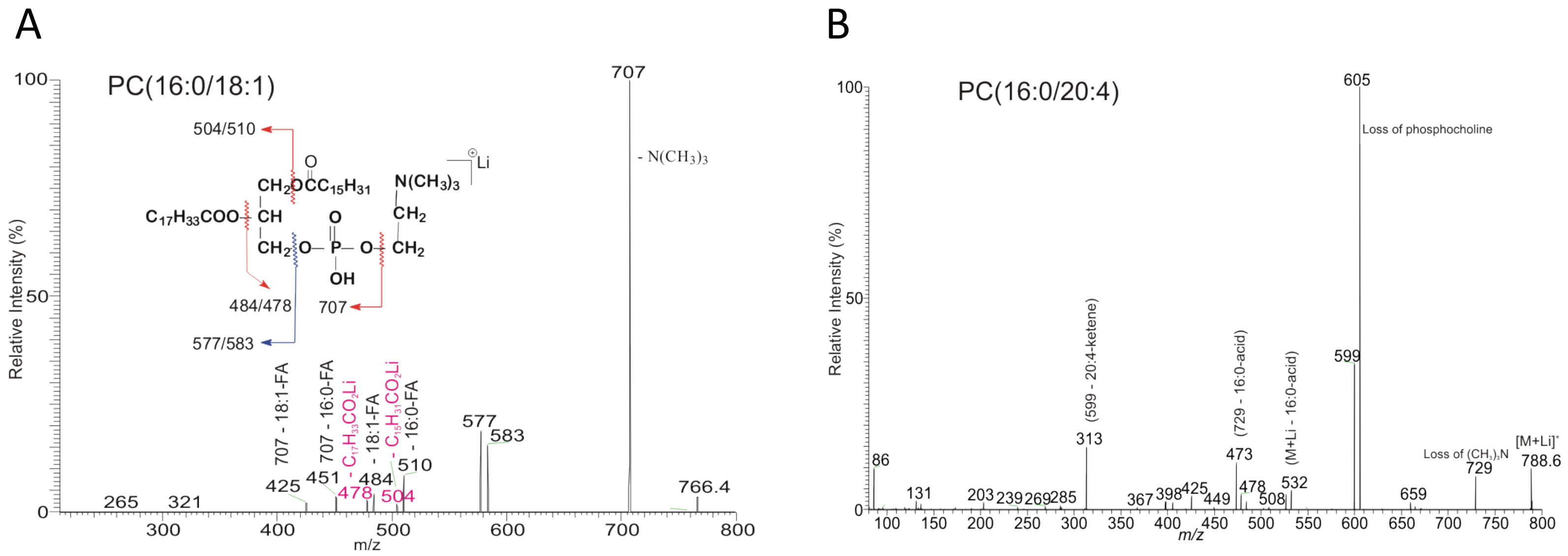

Supplemental Figure S3: Structural assignments of PC. Lipid structures were characterized using a low energy collision induced dissociation (CID) mass spectrometric approach using techniques previously described. MS^2 and MS^3 spectra (each lipid class represented by one to two lipid species) leading to structural assignments. PCs were desorbed and characterized by $[M+Li]^+$ ions by ESI in the positive ion mode using techniques previously described (20-29). The MS^2 spectrum of the $[M+Li]^+$ ions of PC(16:0/18:1) at m/z 766 is shown in (A), and of PC(16:0/20:4) at m/z 788 is shown in (B). The fragmentation pathways leading to the structural assignment is demonstrated in the inset of (A).

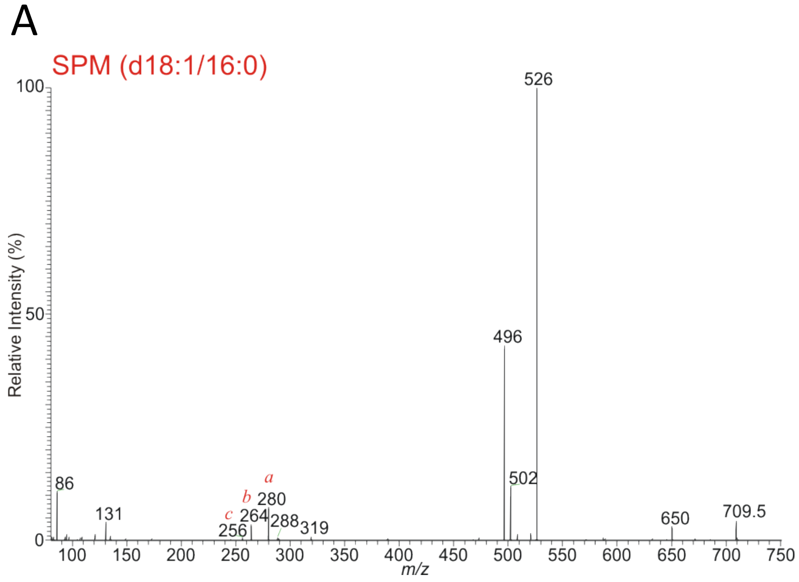

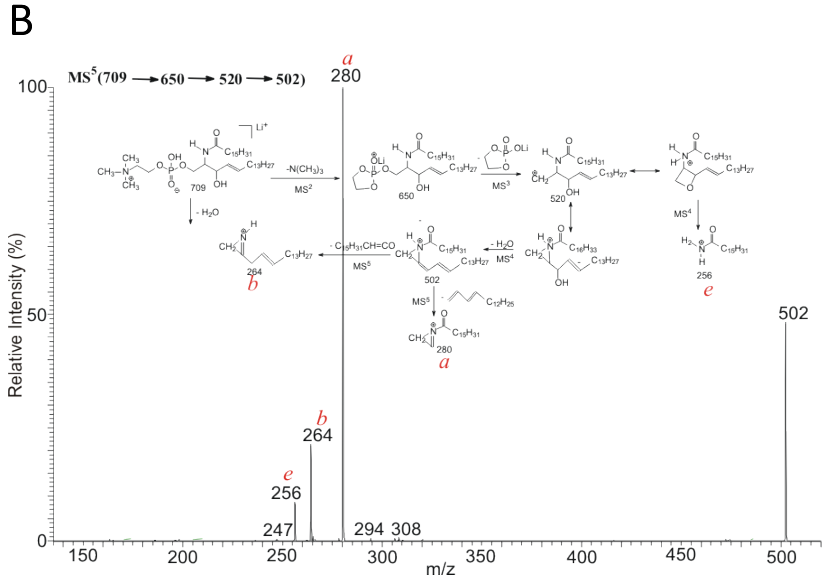

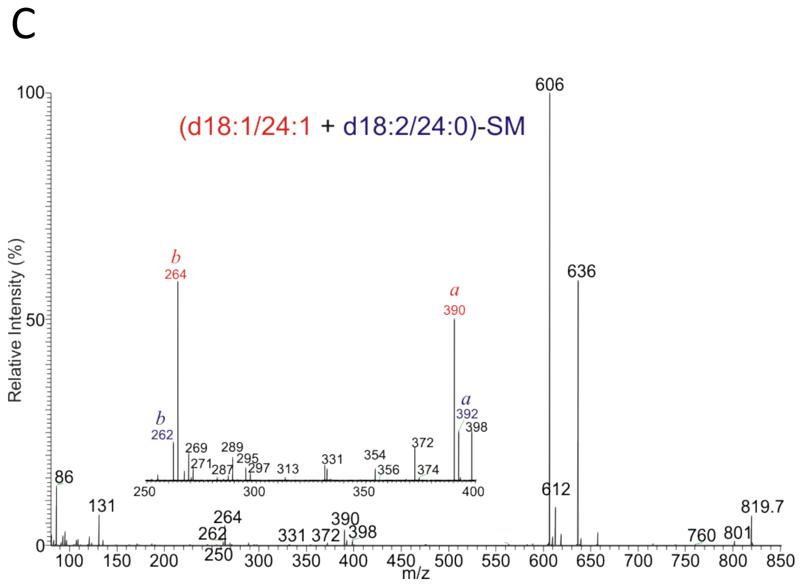

Supplemental Figure S4: Structural assignments of SPM. SPMs were desorbed and characterized by $[M+Li]^+$ ions by ESI in the positive ion mode using techniques previously described (20-29). The MS^2 spectrum of the $[M+Li]^+$ ions of SPM(d18:1/16:0) at m/z 709 (A), and its MS^5 spectrum of the ions of m/z 502 (709→650→520→502). The fragmentation pathways leading to the structural assignment are shown in inset (B). (C) The MS^2 spectrum of the $[M+Li]^+$ ions of m/z 819, representing both a major SPM(d18:1/24:1) and minor SPM(d18:2/24:0) isomers. These assignments were based on the observation of two a/b ion pairs that are also described for SPM(d18:1/16:0) at m/z 709 in (A).

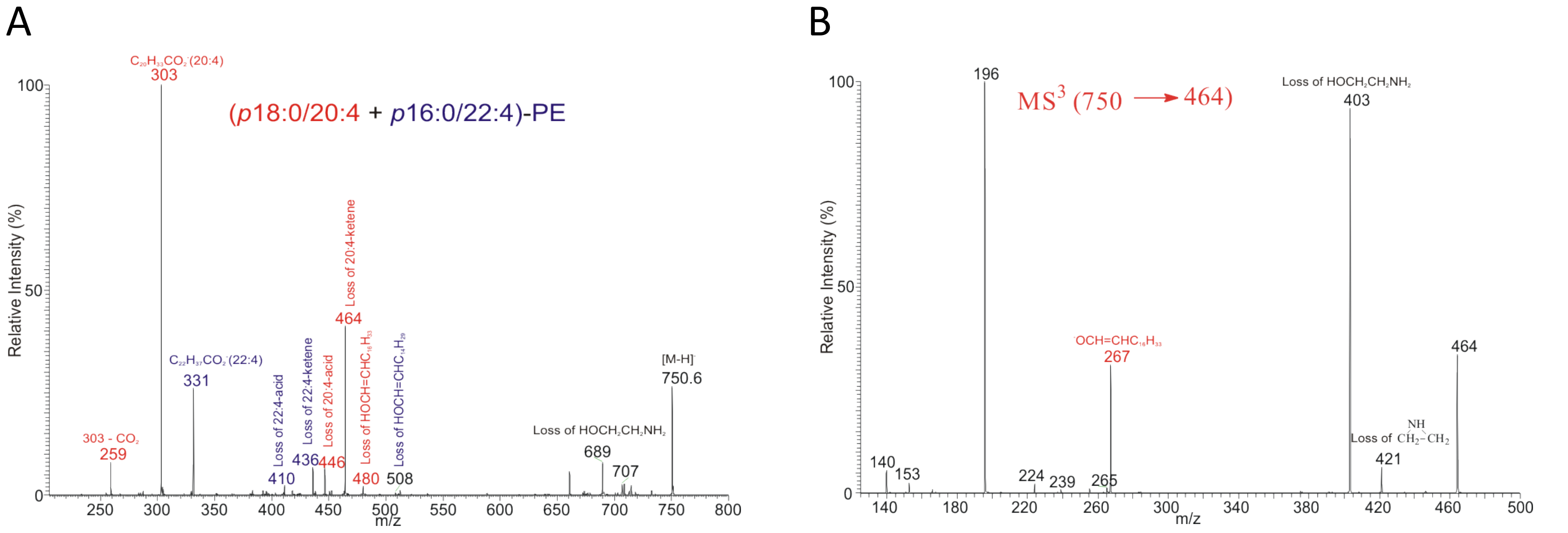

Supplemental Figure S5: Structural assignments of PEs and pPEs. PEs and pPEs were desorbed and characterized by [M – H]$^-$ ions by ESI in the negative ion mode using techniques previously described (20-29). The MS^2 spectrum of the [M - H]$^-$ ions of the ions of m/z 750, representing both a major PE(p18:0/20:4) and minor PE(p16:0/22:4) (A); and its MS^3 spectrum of the ions of m/z 464 (750 → 464) (B), which contains ions at 267. This represents a $C_{16}H_{33}CH{=}CHO^-$ anion that confirms the presence of PE(p18:0/20:4) – a plasmalogen PE.

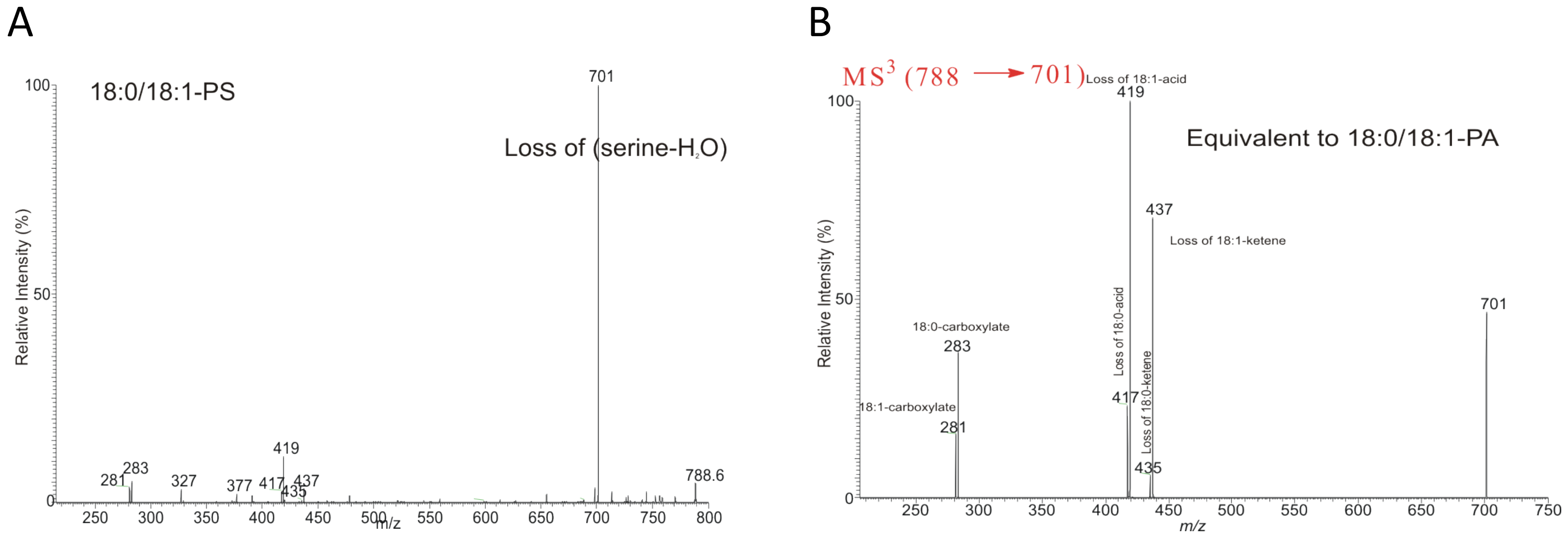

Supplemental Figure S6: Structural assignments of PSs. PSs were desorbed and characterized by $[M-H]^-$ ions by ESI in the negative ion mode using techniques previously described (20-29). The MS^2 spectrum of the $[M - H]^-$ ions of PS(18:0/18:1) at m/z 788 (A), which contains a predominate ion at m/z 701 arising from loss of serine. The MS^3 spectrum of the ions of m/z 701 (788 → 701) (B) contains ions m/z 437/419 from loss of 18:1-FA substituent at sn-2, and the ion pairs at m/z 435/417 arising from loss of 18:0-FA substituent at sn-1, along with ions at m/z 283 and 281 representing 18:0- and 18:1-FA carboxylate anions, respectively. These ions from MS^3 readily led to the structural assignment of PS(18:0/18:1).

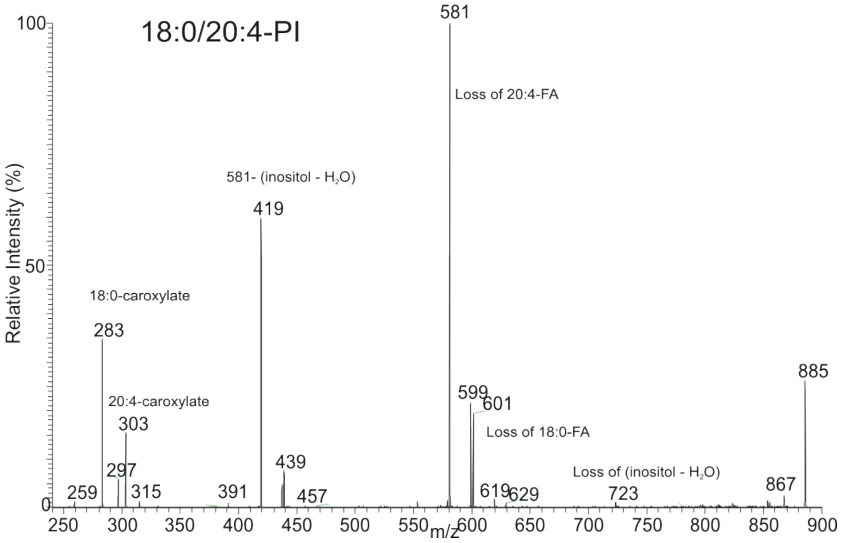

Supplemental Figure S7: Structural assignments of PIs. PIs were desorbed and characterized by $[M – H]^-$ ions by ESI in the negative ion mode using techniques previously described (20-29). The MS^2 spectrum of the $[M - H]^-$ ions of PI(18:0/20:4) at m/z 885, which consists ion pairs at m/z 599/581 from loss of 20:4-FA substituent at sn-2, and the ion pairs at m/z 619/601 arising from loss of 18:0-FA substituent at sn-1, along with ions at m/z 283 and 303 representing 18:0- and 20:4-FA carboxylate anions, respectively. Ions at m/z 419 (581 – inositol + H_2O) and 439 (601 – inositol + H_2O) arose from further loss of inositol, together with 259 representing a phosphoinositol anion ($C_6H_{11}O_5HPO_4^-$), this indicates the presence of the inositol head group. Taken together, these observations led to structural assignment of PI(18:0/20:4).

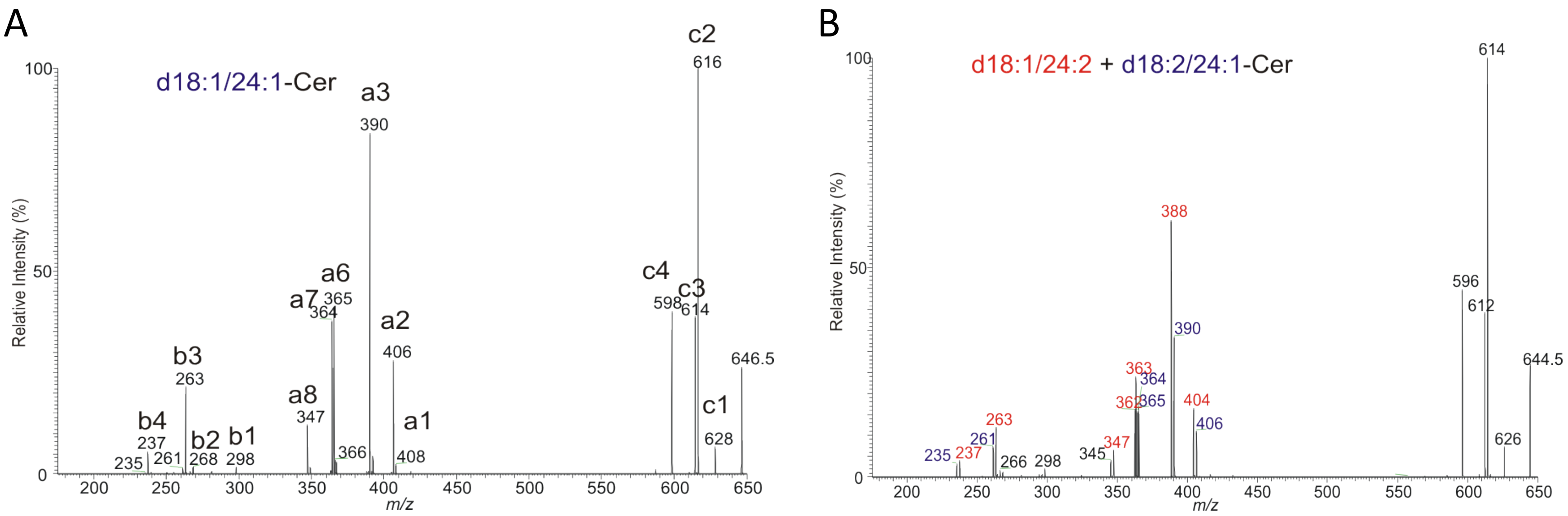

Supplemental Figure S8: Structural assignments of Cers. Cers were desorbed and characterized by $[M - H]^-$ ions by ESI in the negative ion mode using techniques previously described (20-29). The MS^2 spectrum of the $[M - H]^-$ ions of Cer(d18:1/24:1) at m/z 646 (A), and of the ions of m/z 644 (B), represent both major Cer(d18:1/24:2) and minor Cer(d18:2/24:1) isomers. The spectrum contains common ions at m/z 626 (c1) from loss of H_2O, and c2 at m/z 614 (loss of HCHO), c3 at 612 (combined loss of H_2 and HCHO), which are signatures of a ceramide (A). Ions seen at m/z 364 (a6, $C_{23}H_{45}CONH^-$) and 365 (a7, $C_{23}H_{45}CO_2^-$), 408 (a1), 406 (a2), 390 (a3), and 347 (a8)* signifying the presence of 24:1-fatty acyl group, together with ions at 298 (b1), 268 (b2), 263 (b3), 237 (b4)* signifying the presence of 18:1-long chain base led to the structural assignment of Cer(d18:1/24:1). Similar approaches were used to identify the ions for m/z 644 (B). See reference (22) for designation of "a_n", "b_n" and "c_n"; n=1, 2, 3… ions.

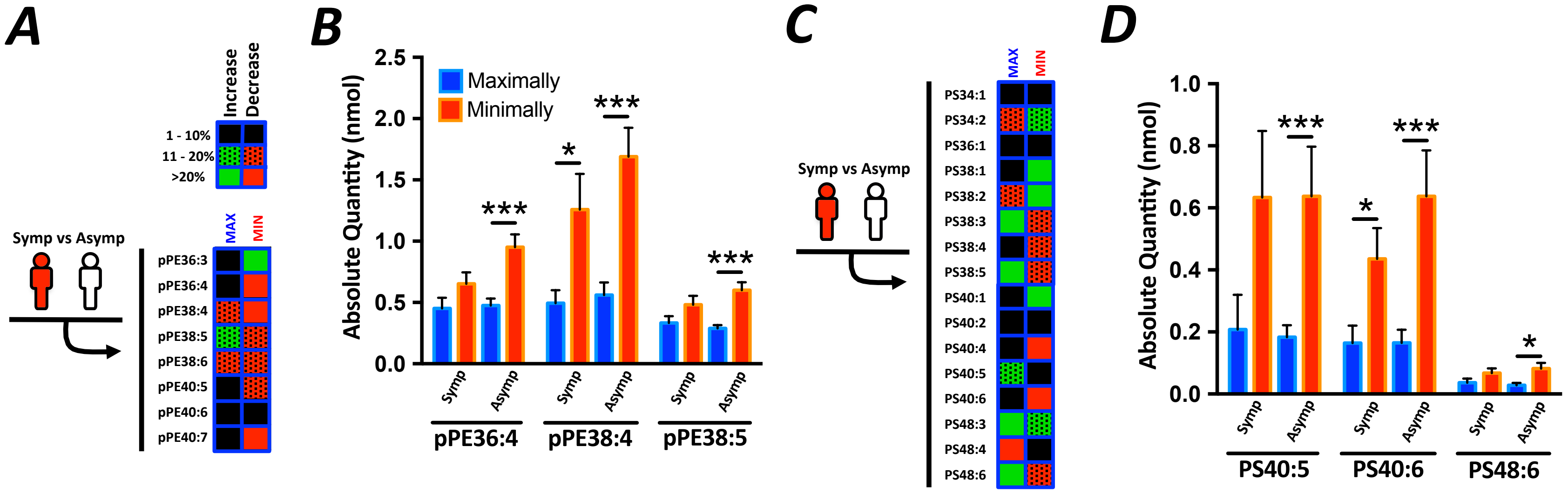

Supplemental Figure S9: Differential pPE and PS content in MAX and MIN diseased CEA segments of symptomatic and asymptomatic subjects. A) Heat map analysis of mass spectrometry lipidomics of MAX and MIN diseased CEA segments from symptomatic (Symp; n = 10) versus asymptomatic (Asymp, n = 7) subjects demonstrates mostly decreased (red) content of pPE species. B) The absolute quantities of pPE36:4, pPE38:4, and pPE38:5 were notably higher in MIN segments. C) Heat map analysis of MAX and MIN segments from Symp versus Asymp subjects demonstrated increased and decreased content of PS species. The absolute quantities of PS40:5, PS40:6, and PS48:6 were higher in MIN segments. * $p < 0.05$ and *** $p < 0.001$.
